# MDA-9/Syntenin small molecule inhibitor IVMT-Rx-4 blocks prostate cancer bone metastasis

**DOI:** 10.1016/j.phrs.2026.108164

**Published:** 2026-03-21

**Authors:** Santanu Maji, Amit Kumar, Padmanabhan Mannangatti, Jinkal Modi, Madeline Gunawardena, Marion Q. LoPresti, Nitai D. Mukhopadhyay, Anne M. Brown, Rudra Pangeni, Qingguo Xu, Webster L. Santos, Jiong Li, Swadesh K. Das, Paul B. Fisher

**Affiliations:** a VCU Institute of Molecular Medicine, Virginia Commonwealth University, School of Medicine, Richmond, VA, United States; b Department of Cellular, Molecular and Genetic Medicine, Virginia Commonwealth University, School of Medicine, Richmond, VA, United States; c Department of Pharmaceutics, Virginia Commonwealth University, Richmond, VA, USA; d Department of Biochemistry, Virginia Tech, Blacksburg, VA, USA; e Department of Biostatistics, Virginia Commonwealth University, Richmond, VA, USA; f VCU Massey Comprehensive Cancer Center, Virginia Commonwealth University, School of Medicine, Richmond, VA, United States; g Department of Chemistry, Virginia Tech, Blacksburg, VA, USA; h Department of Medicinal Chemistry, School of Pharmacy, Virginia Commonwealth University, Richmond, VA, United States

**Keywords:** MDA-9/Syntenin, BM-MSC, PDGF-AA, CXCL5 and NF-κB

## Abstract

Bone metastasis is a frequent and incurable consequence of advanced prostate cancer (PC). This process originates through an interplay between disseminated tumor cells and heterogeneous bone resident cells in the metastatic niche. *Melanoma differentiation associated gene-9* (*mda-9/Syntenin*) is a pro-metastatic gene expressed in multiple organs, including bone marrow-derived mesenchymal stromal cells (BM-MSCs), under both physiological and pathological conditions. MDA-9/Syntenin coordinates the interactions between tumor cells and BM-MSCs, which promote establishment of metastatic tumors in the bone niche. Considering the importance of protein-protein interactions in regulating MDA-9/Syntenin functions, we focused on developing small molecule inhibitors of these interactions. We describe the translational potential of IVMT-Rx-4, an intermediate synthesis product of PDZ1i, in inhibiting PC bone metastasis. IVMT-Rx-4 has similar bioactivity as PDZ1i but with improved druggable properties, e.g., higher solubility and lower efflux. It promotes potent anti-invasive and anti-metastatic effects by inhibiting the MDA-9/Syntenin dependent tumor-derived platelet derived growth factor, PDGF-AA, and its related signalling in BM-MSCs. In addition, the combination of IVMT-Rx-4 and docetaxel enhances survival in experimental bone metastasis models. These observations reinforce the concept that together with metastasis suppression, IVMT-Rx-4 can boost the effectiveness of standard-of-care treatment. Collectively, the present work provides a framework for translational strategies to ameliorate health complications and morbidity associated with advanced PC.

## 1. Introduction

The metastatic growth of prostate cancer (PC) within bones, the most advanced stage of this disease is frequently associated with skeletal complications and worsening of quality of life [1]. Rather than palliative or pain management options, there are no curative treatments currently available [2]. Consequently, current strategies only partially manage this disease that ultimately results in a decreased quality of life and mortality. Bone metastasis is an intricate process in which the tumor cell resides in the bone environment and forms close physical and functionalconnections with the adjacent stromal components [3]. These interactions promote the growth of the tumor and disrupt the tightly regulated homeostasis, i.e., the fine balance between bone formation and resorption activities leading to bone mass gain and/or developing porous bones [4,5]. Accordingly, comprehending the metastatic process, identifying the crucial genes that functionally contribute to tumor cell dissemination and that interact with stromal cells to facilitate tumor growth and ultimately developing therapeutic targeting strategies for these tumor cells are fundamentally important objectives for enhanced therapy of PC.

Melanoma differentiation associated gene-9 (MDA-9/Syntenin), also known as Syndecan Binding Protein (SDCBP), or Syntenin-1 (referred to as MDA-9/Syntenin) was initially cloned by us [6,7] and is primarily categorized as a pro-metastatic gene in various tumor types, including PC [4,7,8]. Experimental data continues to refine our understanding of MDA-9/Syntenin multifunctional activities implicated in the metastatic process, e.g., invasion and migration, epithelial mesenchymal transition, angiogenesis, immune escape, since its initial description (reviewed in [8]). It is now established that diverse cancers display robust tumor-specific MDA-9/Syntenin expression that positively correlates with poor prognosis, thereby emphasizing its potential as a molecular target for developing anti-metastatic/tumor therapy [9–13]. Moreover, the tumor cell extrinsic function of MDA-9/Syntenin and its impact on disease progression has also been documented. MDA-9/Syntenin modulates exosome composition and influences the exosome-mediated angiogenesis process in breast cancer. The absence of MDA-9/Syntenin in stromal cells markedly affects the metastatic proliferation of melanoma and prostate cancer in the lung [14] and bone [4], respectively. It also modulates the expression of several cytokines by activating the NF-κB or STAT pathways, thereby influencing the microenvironment and promoting tumor growth [15]. Despite its crucial role in cancer causation and normal physiological functions, MDA-9/Syntenin is not essential for survival. No physiological impairment was seen in global knockout animals, suggesting that targeting will not be deleterious [14, 16]. Our most recent findings accurately elucidate the tumor-intrinsic and -extrinsic functions of MDA-9/Syntenin in the progression of prostate cancer bone metastasis [4]. These data collectively underscore the potential of MDA-9/Syntenin protein as a targetable molecule for impeding PC bone metastasis, and in principle other sites of metastases.

MDA-9/Syntenin forms a functional and stable complex by interacting with partner protein(s) through its two tandem PDZ domains (PDZ1 and PDZ2), thereby activating specific transcription factors, e.g., NF-κB [12], STAT3 [9], etc. The integrity of both PDZ domains is critical to MDA-9/Syntenin function, as targeting a single domain using genetic or pharmacological inhibitors is sufficient to disrupt activity [10]. Considering the similarity of PDZ domains in numerous scaffold proteins and their involvement in critical physiological functions, PDZ domains were previously considered ‘non-druggable’. Using fragment-based drug-discovery (FBDD) guided by *in silico* docking and NMR-informed design, we generated a first-in-class PDZ1 (first PDZ domain from the N-terminal end of MDA-9/Syntenin) inhibitor targeting small molecule (PDZ1i) with potent anti-invasive and anti-metastatic properties in glioblastoma [11], prostate [17], neuroblastoma [18] and breast cancer [15] pre-clinical animal models. IVMT-Rx-4, an intermediate component in the synthesis of PDZ1i, displays similar bioactivities with improved solubility, lower efflux and simple/rapid/inexpensive synthesis and has now been assessed for pharmacological properties, *in vitro* activities, and *in vivo* efficacy against PC bone metastasis.

## 2. Methods and materials

### 2.1. Synthesis and docking model of IVMT-Rx-4

IVMT-Rx-4 was synthesized by WuXi Apptec, Inc., based on the structure described by us in Kegelman *et al.* [11]. The synthesis scheme, HPLC and NMR data for the final product is provided in a Supplementary file (Synthesis report). The computational docking model was developed using GNINA v1.1 [19] and Glide from Schrodinger Suite version 2024–4 [20] and the methods description is provided in a Supplementary file (Methods for IVMT-Rx-4 Docking).

### 2.2. Cell lines

Cell lines, both human and murine origins, used in this study were previously described [4,9,17]. Briefly, the human bone marrow-derived mesenchymal stromal cell line HS5, prostate cancer cell lines LNCaP and VCaP were purchased from ATCC and maintained as described by ATCC. The luciferase expressing bone metastatic cell line PC3-ML (PC3-ML-*Luc*), was provided by Dr. M. G. Pomper (UT Southwestern Medical School, Dallas, TX) [21]. The murine prostate cancer cell line RM1 and its variant clone isolated from the bone niche, RM1-BM-*Luc*, were obtained from Dr. T. C. Thompson (Baylor College of Medicine in Houston, TX) [22]. ARCaP-M cells were purchased from Novicure Biotechnology (Birmingham, AL) and cultured using MCaP medium following instructions from Novicure, Inc. USA. C4–2B was obtained from Dr. K. Dutta (Virginia Commonwealth University, School of Medicine, Richmond, VA). All cell lines were routinely examined for mycoplasma contamination using commercial mycoplasma detection kits.

### 2.3. Target engagement assay

The assay was developed based on published literature [23] and conducted by a Contract Research Organization, Pharmaron, Inc., following the SOP. The protocol is provided in a Supplementary file.

### 2.4. Colony formation assay

200 cells/well were seeded in triplicate in 6-well plates. After overnight incubation, cells were treated with DMSO (0.5%) or IVMT-Rx-4 (50 μM) three times during a 2-week period. The colonies were fixed with ice-cold Methanol and stained with 0.5% crystal violet and counted [12].

### 2.5. Real-time PCR

Total RNA was extracted from cells using miRNeasy kits (Qiagen), and cDNA was prepared as described previously [4]. Gene specific TaqMan Probes were obtained from Applied Bio system, Thermo-Fisher, USA. qRT-PCR was performed using an ABI Quant Studio real-time PCR.

### 2.6. Invasion assays

Boyden chamber assays were performed using Corning^®^ BioCoat^®^ Matrigel^®^ Invasion Chambers obtained from Corning Inc., USA. In brief, cells were seeded on the Matrigel-coated upper chamber and treated with IVMT-Rx-4. Invaded cells were fixed, stained and counted after 18 hrs. as previously described [17].

### 2.7. Antibodies

MST1, pMST1, YAP, phospho-YAP, β-actin, PDGFR-α, phospho-PDGFR-α antibodies were purchased from Cell Signaling Technology, USA. Docetaxel was obtained from Sigma Aldrich, USA. Recombinant human PDGF-AA was purchased from R&D Biosystem, USA.

### 2.8. ELISA

Human and mouse specific CXCL5, PDGF-AA ELISA kits were obtained from R&D Systems, USA. All ELISAs were performed using the manufacturer’s protocol.

### 2.9. NF-κB activation assay

NF-κB p65 transcription factor assay kits were obtained from Cayman Chemical, USA and performed following the manufacturer’s instructions. First, cells were treated with IVMT-Rx-4 for 24 hrs. and then NF-κB p65 transcription factor assay was performed according to a previously described protocol [24].

### 2.10. Bone marrow derived mesenchymal stromal cell isolation

Total bone marrow cells were collected from 6 to 8 weeks male *MDA-9/Syntenin*^WT^ (*mda-9*^WT^) and global *MDA-9/Syntenin* knockout (*mda-9*^−/−^) mice as previously described in Das *et al*. [14]. *mda-9*^WT^ and *mda-9*^−/−^ bone marrow derived mesenchymal stromal cells were isolated using a previously outlined protocol [4,25].

### 2.11. Animal studies

All in vivo experiments were performed in strict accordance with the guidelines and regulations approved by the Institutional Animal Care and Use Committee (IACUC) at Virginia Commonwealth University (VCU), IACUC protocol number No. AM10183, and comply with ARRIVE (Animal Research: Reporting of In Vivo Experiments) guidelines. Since prostate cancer develops in the male reproductive organ, to study the therapeutic efficacy of IVMT-Rx-4 we used only male animals.

#### 2.11.1. Intracardiac cancer cell implantation

For experimental bone metastasis experiments, 3 × 10^4^ RM1-BM-*Luc* cells were injected by the intracardiac route to develop bone-metastases in 6- to 8-week-old male C57BL/6 mice as described previously [4]. Incidence of metastasis was monitored using Bioluminescence imaging, as described previously [4,26].

#### 2.11.2. Survival studies

Both syngeneic (C57BL/6) and athymic nude mice were used for survival studies. Tumor cells (RM1-BM-*Luc* or PC3-ML-*Luc*) were injected intracardially in a cohort of 40 animals. Mice were randomly divided into four groups (n =10), a) control, b) IVMT-Rx-4, c) Docetaxel (Sigma-Aldrich, USA) and d) combination. Mice received intraperitoneal IVMT-Rx-4 (30 mg/kg body weight) or Docetaxel (5 mg/kg body weight) 3X a week for two-weeks only. In the combination groups, both regimens were administered 3X a week for two-weeks at the same dose as a single therapy. Animals were imaged periodically and maintained until euthanization was required.

### 2.12. Statistical analysis

Data are represented as the mean ± SEM and analyzed for statistical significance using the student t test or ANOVA test in comparison with corresponding controls followed by Newman-Keuls test as a post hoc test. P < 0.05 was considered as statistically significant. For survival studies, Cox Proportional hazards survival regression was analyzed using GraphPad Prism software. Treatment specific metastasis probabilities were estimated using point estimate and 95% confidence interval estimate using a binomial model.

## 3. Results

### 3.1. Synthesis and primary characterization of IVMT-Rx-4, an MDA-9/Syntenin antagonist

**Fig. 1.**
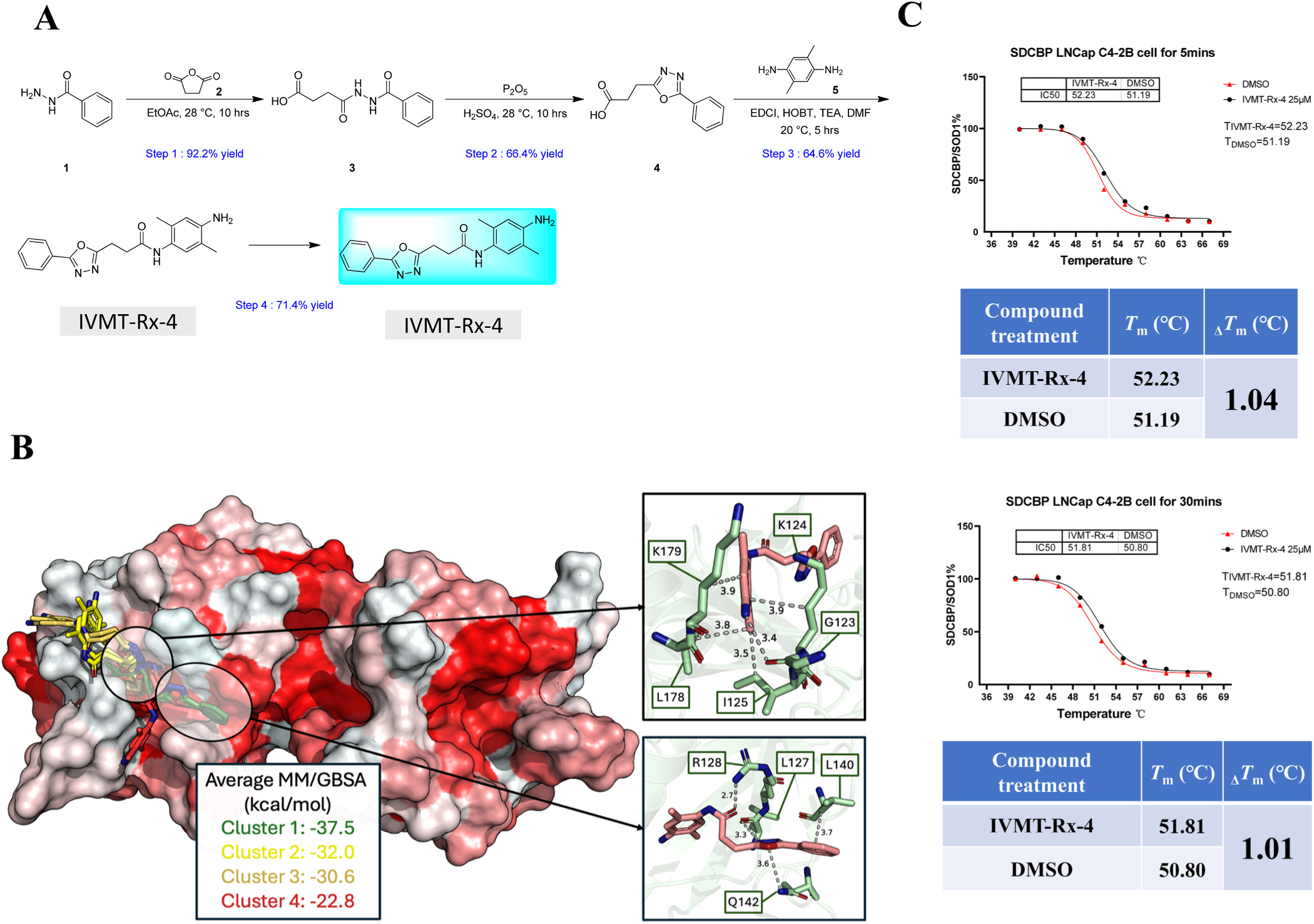

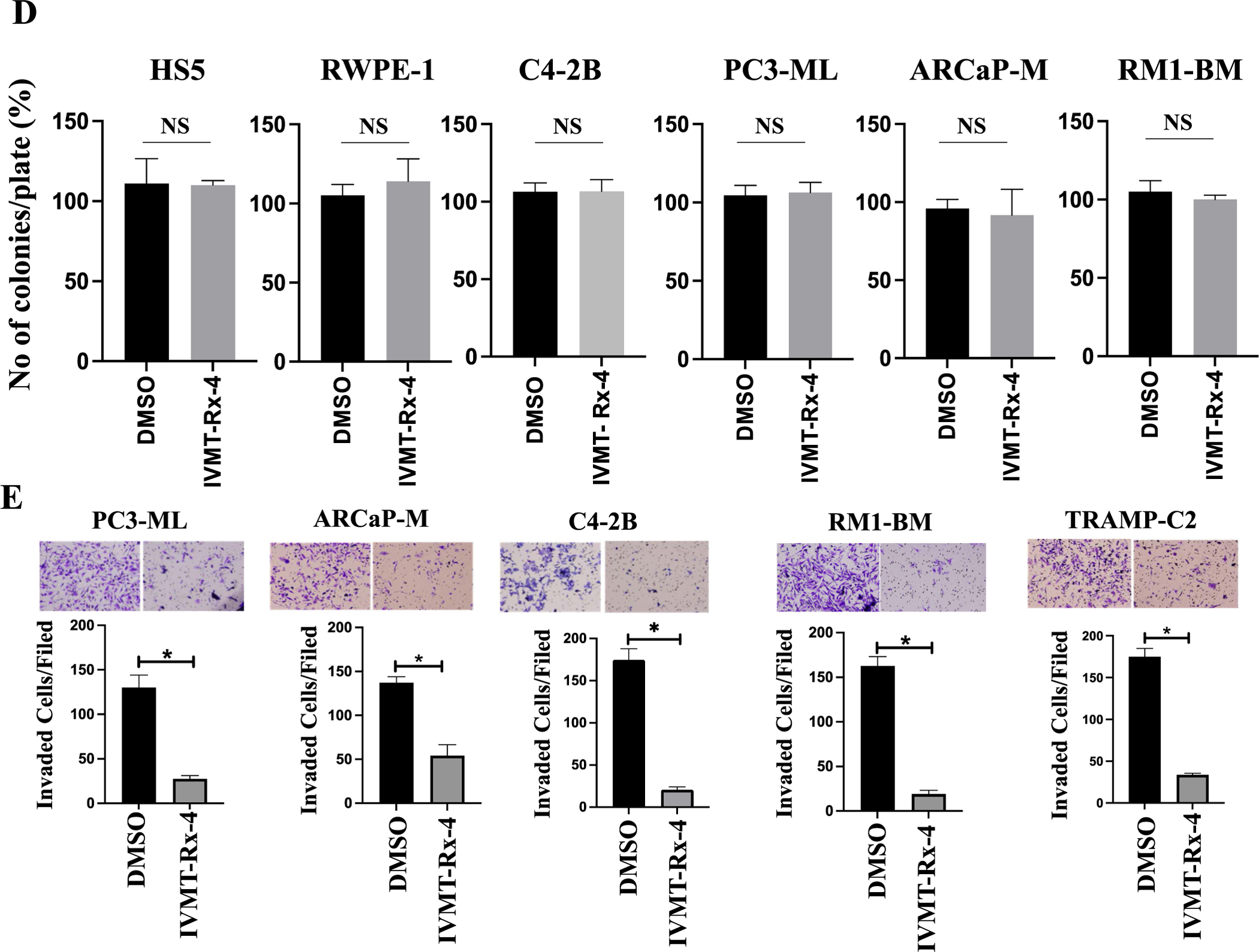
Primary characterization of IVMT-Rx-4. A) Chemical structure (upper panel) and synthesis scheme (lower panel). B) Docking model of IVMT-Rx-4 and the PDZ1 region of MDA-9. C) Cellular thermal shift assay evaluating drug (IVMT-Rx-4) target (MDA-9/Syntenin) interactions in C4–2B cells. After being exposed to either a control vehicle or 25 μM IVMT-Rx-4 for 5 min, C4–2B cells were heated at various temperatures for 3 min. All proteins were extracted, and Western blotting was performed immediately after heating. Quantifications were obtained in the absence (control) or presence of IVMT-Rx-4 and values are presented as percentage of RT (room temperature) normalized for the experimental groups (control, IVMT-Rx-4). SOD (Superoxide dismutase) was used as an endogenous control. D) HS5, RWPE-1, C4–2B, PC3-ML, ARCaP-M and RM1-BM cells (250 cells/well) were seeded in 60-mm culture plates and treated with either DMSO or 50 μM of IVMT-Rx-4 for 10 days (Every alternate day the media was replaced with fresh media containing DMSO or IVMT-Rx-4). Colonies were stained with crystal violet and counted (n = 3 ± SD; ns: non-significant). E) PC3-ML, ARCaP-M, C4–2B, RM1-BM and TRAMP-C2 cells were seeded in the upper chamber of the insert and treated with either DMSO or 25 μM of IVMT-Rx-4 for 18 hrs. Invaded cells were stained with crystal violet. Top panel: representative photograph of invaded cells under different experimental settings. Bottom panel: graphical representation of number of invaded cells/field for different cell lines (n = 3 ± SD; * p < 0.05).

IVMT-Rx-4 is a synthetic intermediate of our previously reported MDA-9/Syntenin antagonist PDZ1i [11]. The synthesis scheme (Fig. 1A) (the chemical characterization data are described in Supplementary Document) and the projected docking model is described in Fig. 1B, respectively. More comprehensive analysis is described in Supplementary files (Supplementary Fig. S1A-D). In brief, docking results identify four primary clusters of IVMT-Rx-4 binding poses (Fig. 1B-Upper sub panel). Across all clusters, an aromatic moiety is consistently located between residues K124 and K179, highlighting the importance of this hydrophobic interaction site. In the predicted lowest free energy pose, L178 is positioned adjacent from the methyl group on the benzene ring, while I125 is perpendicular in orientation (Fig. 1B-Lower sub panel). The hydrophobicity and depth of this pocket suggests that the placement of the aromatic moiety is critical for stable binding. We used cellular thermal shift assays (CETSA) [23] and Western blotting (Supplementary Fig. S2) to validate the binding of IVMT-Rx-4 with MDA-9/Syntenin in intact cells. As shown in Fig. 1C, in C4–2B cells, IVMT-Rx-4 treatment enhanced the melting (*T*_*m*_) point of MDA-9/Syntenin to a higher temperature (at 5 and 30 mins after treatment, 52.23ºC and 51.81ºC, respectively) compared to corresponding DMSO treated group (at 5- and 30-min time points, 51.19ºC and 50.80ºC, respectively), suggesting a drug-target engagement in cells. Although the alterations in thermal stability are low in intensity, it displays long-term persistence (at least 30 min) indicating a considerable engagement to ensure a sustained blocking of MDA-9/Syntenin’s ability to interact with partner proteins.

IVMT-Rx-4 is solid with high crystallinity. Multiple thermal events were observed in the DSC curve (Supplementary Table S1), indicating phase transitions upon heating. It was slightly hygroscopic, and the moisture adsorption at 80/90% RH was 0.98/1.06%. The crystal form remained unchanged after DVS testing. PSD testing showed that the average value of Dv [90] was 58.6 μm. It showed relative high solubility in SGF and low solubility in FaSSIF, FeSSIF and SIF. The solubility of IVMT-Rx-4 is pH dependent, and the solubility increased with a decrease of pH (Supplementary Table S2). A new pattern was obtained in SGF and pH 1.2 buffer after the solubility test. However, degradation was observed in both media. IVMT-Rx-4 showed relatively good solubility in MeOH, EtOH, CH_2_Cl_2_, Acetone and MeCN, low solubility was observed in water, and it was almost insoluble in hexane. New patterns were observed during solubility measurement in organic solvents. A detailed description with primary data is available in Supplementary files under the “Solid phase characterization” section.

### 3.2. Inhibition of PC invasion by IVMT-Rx-4

With few exceptions, MDA-9/Syntenin expression and function are not critical for cell viability, as evidenced by *in vitro* knockout studies [9] and observations from global knockout mice [14]. Our prior research, employing PDZ1i, substantiates this concept [17]. The present work initially investigated using clonal assay shows the effects of IVMT-Rx-4 on both human and murine PC cell lines, as well as on bone marrow-derived mesenchymal stromal cells (HS5). In both tumor and non-tumor cells, six repeated doses over a two-week period with 50 μM IVMT-Rx-4 or PDZ1i did not inhibit cell proliferation (Fig. 1D), thereby reinforcing the non-cytotoxic characteristics of the MDA-9/Syntenin antagonists (PDZ1i [17] and IVMT-Rx-4).

The effects of MDA-9/Syntenin on cellular invasion are well documented [9,17]. To investigate the functional impact of pharmacological inhibition of MDA-9/Syntenin, we assessed the effectiveness of IVMT-Rx-4 in a Transwell invasion assay. IVMT-Rx-4 effectively suppressed cellular invasion at a concentration of 25 μM in all five cell lines, including human (ARCaP-M, PC3-ML, C4–2B) and two murine PC cell lines (RM1-BM and TRAMP-C2) (Fig. 1E). It is worth mentioning that IVMT-Rx-4 did not demonstrate any superior biological efficacy compared to PDZ1i (Supplementary Fig. S3). However, when we evaluated the solubility (Thermodynamic solubility in PBS, pH 7.4; 160 mg/ML vs 10 mg/ML, for IVMT-Rx-4 vs PDZ1i) and Caco-2 permeability (Efflux ratio 0.92 vs 300, for IVMT-Rx-4 vs PDZ1i), IVMT-Rx-4 exhibited greater efficacy compared to PDZ1i (Table 1, Experimental details are described in Supplementary files under ADME/PK sections), which prompted us to further investigate therapeutic potential of this small molecule in more detail *in vivo*.

ADME data with IVMT-Rx-4 indicates no inhibitory effects on most of the members of the CYP family of proteins (except CYP2C8, which is a less common CYP), no time-dependent inhibition when tested with CYP4A, and acceptable values achieved in hERG assays. Based on favorable ADME, (enhanced solubility and lower efflux), IVMT-Rx-4 may provide an appropriate MDA-9/Syntenin PDZ1 small molecule inhibitor for further development (Table 2, Experimental details are described in Supplementary files under ADME/PK sections (conducted by a Contract Research Organization, Pharmaron, Inc.).

Mean plasma concentration versus time profiles and the pharmacokinetic parameters of IVMT-Rx-4 following a single-dose administration via IV (3 mg/kg), PO, SC, and IP routes (10 mg/kg each) are shown in Supplementary Table S3-S6 (independently with the route of administration). Maximum plasma concentration (C_max_) values for IV, PO, SC, and IP administration were 2475, 2047, 1017, and 1012 ng/ML, respectively. The time to reach C_max_ (T_max_) for PO, SC, and IP were 0.25, 0.50, and 0.25 h, respectively. Plasma levels of IVMT-Rx-4 declined steadily and became undetectable after 8 h for PO, SC, and IP routes, and after 4 h for IV administration (Supplementary Fig. S4). Plasma half-lives were 0.45 h (IV), 0.69 h (SC), 1.37 h (PO), and 1.02 h (IP).

### 3.3. IVMT-Rx-4 inhibits NF-κB activation and PDGF-AA expression in different PC cell lines

**Fig. 2.**
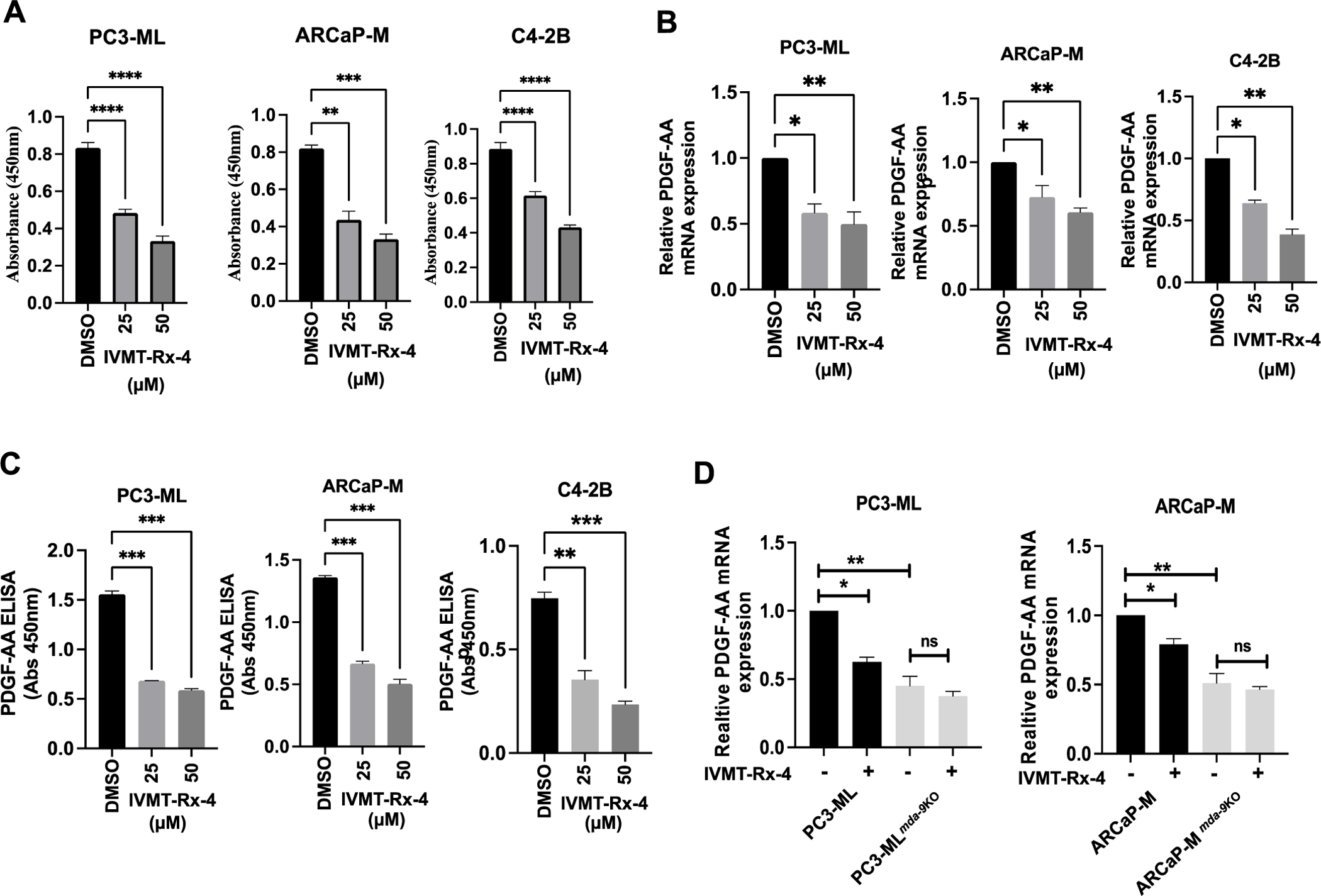
IVMT-Rx-4 downregulates PDGF-AA expression. A) Three different PC cell lines (PC3-ML, ARCaP-M and C4–2B) were treated with the indicated doses of IVMT-Rx-4 for 24 hrs. NF-κB activity was measured by absorbance at 450 nm (n = 3 ± SD; * p < 0.05). B) *PDGF-AA* mRNA expression was determined in the three PC cell lines after 24 hrs. treatment with 25 μM or 50 μM IVMT-Rx-4. C) PDGF-AA protein levels were determined in the three PC cell line-derived conditioned media by ELISA. In both panels: n = 3 ± SD; * p < 0.05. D) Control PC3-ML and ARCaP-M cells and respective *mda-9* knockout cells were treated with IVMT-Rx-4 for 24 hrs. and *PDGF-AA* mRNA expression was analyzed by qRT-PCR (n = 3 ± SD; * p < 0.05).

The physical interaction between MDA-9/Syntenin and its companion proteins activates different transcription factors and effector genes, thereby promoting the metastatic phenotype [10,15,24]. Our recent research revealed that the MDA-9/Syntenin/NF-κB axis stimulates the synthesis of PDGF-AA [4], a growth factor that facilitates the progression of bone metastases in PC. Based on these considerations, we investigated whether IVMT-Rx-4 could influence NF-κB activity. In comparison to the vehicle-treated group, IVMT-Rx-4 decreased NF-κB activity in all three established PC lines associated with bone metastasis (Fig. 2A). We verified that IVMT-Rx-4 decreased PDGF-AA at both the mRNA (Fig. 2B) and protein levels (Fig. 2C), validating the functional consequences of NF-κB inhibition (Fig. 2A). We next evaluated *mda-9* knockout clones (e.g., PC3-ML^*mda−9 KO*^ and ARCaP-M^*mda−9 KO*^, developed and characterized previously [9]) to establish *mda-9* target specificity. As anticipated, the expression of PDGF-AA was diminished in both clones relative to the basal level in the parental line. This supports the role of MDA-9/Syntenin in this pathway. Nonetheless, IVMT-Rx-4 treatment did not further reduce PDGF-AA levels in *mda-9* knockout clones, thereby confirming this inhibitor's selectivity for *mda-9* at a cellular level (Fig. 2D).

### 3.4. IVMT-Rx-4 inhibits CXCL5 expression in bone marrow derived mesenchymal stromal cells

**Fig. 3.**
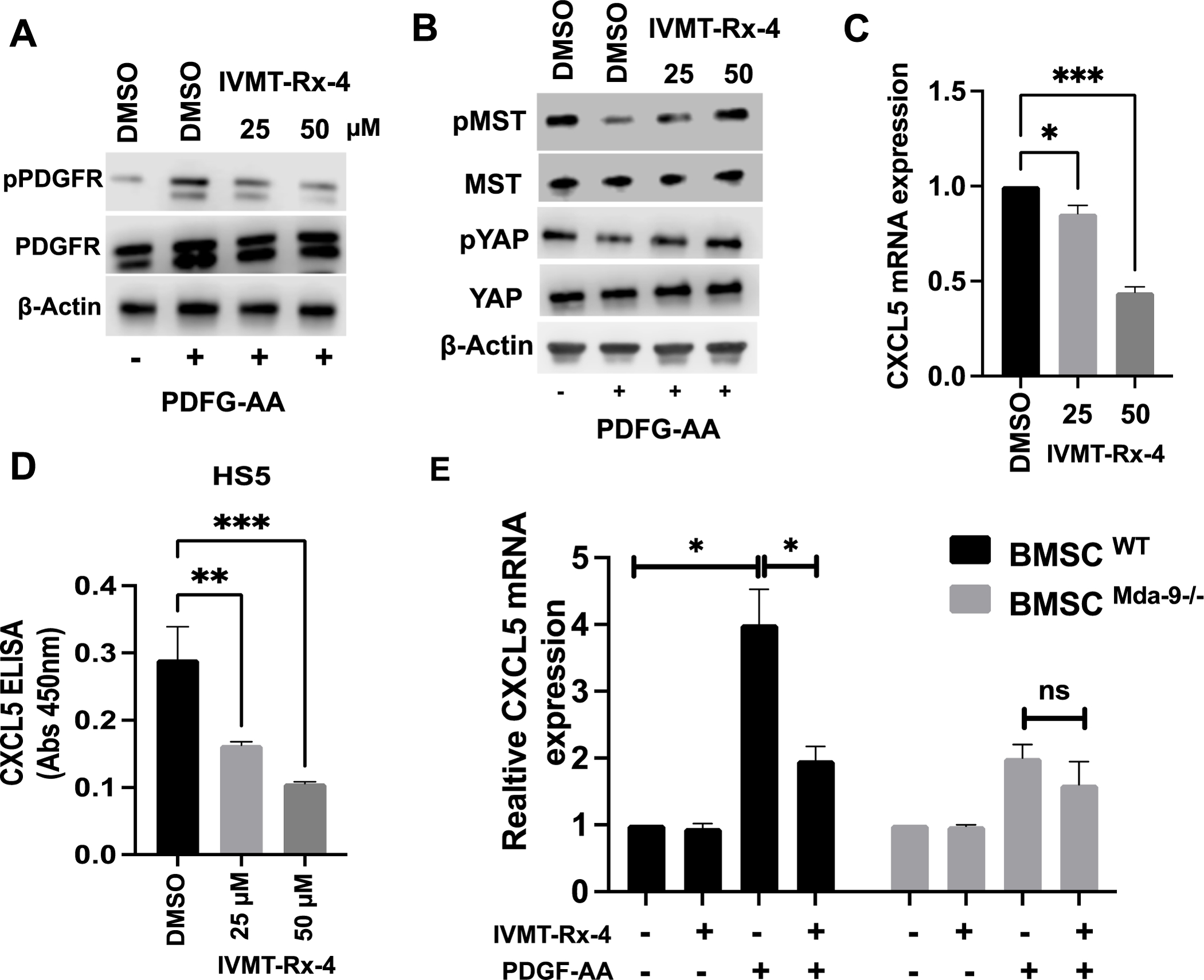
IVMT-Rx-4 blocks PDGF-AA stimulated CXCL5 expression in BM-MSCs. A and B) Human bone marrow derived mesenchymal stromal cells (HS5) were cultured with recombinant PDGF-AA (10 ng/ML) in the presence or absence of 25 μM IVMT-Rx-4 for 24 hrs. and equal amounts of cell lysates were subjected to Western blotting with the indicated antibodies. C) HS5 cells were treated with the indicated doses of IVMT-Rx-4 for 24 hrs. *CXCL5* mRNA expression was measured by qRT-PCR. D) HS5 cells were treated with the indicated doses of IVMT-Rx-4 and after 24 hrs. the conditioned media (CM) was collected. Secreted CXCL5 in CM was measured by ELISA (n = 3 ± SD; * p < 0.05). E) Bone marrow derived mesenchymal stromal cells were isolated from WT and *mda-9* KO C57BL/6 mice and treated with recombinant murine PDGF-AA (10 ng/ML) in the absence or presence of 25 μM IVMT-Rx-4 for 24 hrs. CXCL5 mRNA expression was measured by qRT-PCR (n = 3 ± SD; * p < 0.05).

BM-MSCs are vital for the expansion of bone metastases inside the osseous microenvironment. Previous studies demonstrate that BM-MSCs expresses MDA-9/Syntenin, and abrogation of its expression impedes bone metastasis [5]. PDGF-AA significantly contributes to tumor growth by interacting with PDGFRα and modulating the Hippo pathway. CXCL5 serves as the downstream effector, and our experimental evidence confirms its relevance in the progression of bone metastases [5]. To recapitulate the signaling in the bone niche, we activated HS5 with recombinant PDGF-AA in the presence or absence of IVMT-Rx-4. PDGF-AA induced activation of the PDGFRα receptor; however, IVMT-Rx-4 treatment inhibited this activation, as seen by the downregulation of phosphorylated PDGFRα (Fig. 3A). Consistent with IVMT-Rx-4's capacity to inhibit PDGFR activation, this MDA-9/Syntenin antagonist promoted the phosphorylation of MST and subsequently the phosphorylation of YAP at ser127 (Fig. 3B). The phosphorylation of YAP causes its cytoplasmic accumulation and inability to activate transcription, leading to the downregulation of CXCL5. IVMT-Rx-4 significantly decreased the mRNA and protein levels of CXCL5 (Fig. 3C and Fig. 3D) in PDGF-AA activated HS5 cells. Subsequently, we reaffirmed the target specificity of IVMT-Rx-4 utilizing freshly isolated murine BM-MSCs from *mda-9* knockout animals [4,14]. Murine PDGF-AA was used to stimulate mBM-MSCs in conjunction with IVMT-Rx-4, and the expression of *CXCL5* mRNA was quantified at a single time point. The results indicated that CXCL5 suppression occurred exclusively in mBM-MSC^*WT*^ and not in mBM-MSC^*mda−9 KO*^ cells (Fig. 3E), hence verifying the specificity of IVMT-Rx-4.

### 3.5. IVMT-Rx-4 inhibits experimental bone metastasis and enhances animal survival

**Fig. 4.**
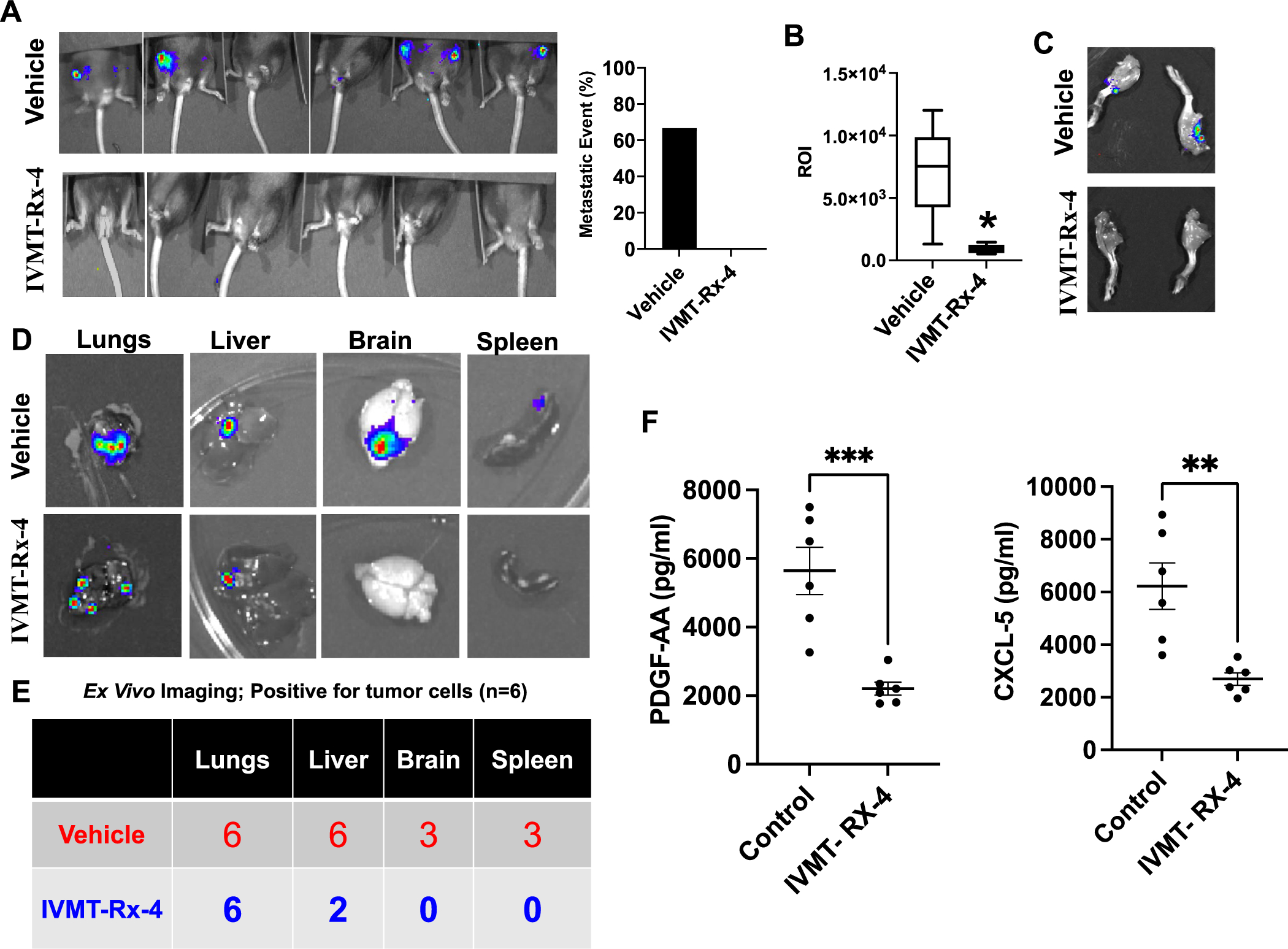
IVMT-Rx-4 promotes anti-bone metastatic effects. RM1-BM-*Luc* cells were injected through the intracardiac (I.C.) route into C57BL/6 mice to establish experimental bone metastases. Vehicle (DMSO) or IVMT-Rx-4 (30 mg/kg body weight) were administrated intraperitoneally (I.P.) 3X a week for two weeks (total 6 doses). A) BLI imaging was performed on day 15 and the number of bone metastasis positive mice were graphically represented as metastatic events. B) Calculated ROI values are graphically represented (n = 6 ± SEM; * p < 0.05). C) *Ex vivo* BLI image of representative bones from both vehicle and IVMT-Rx-4 treated animals. D) Representative *ex vivo* images and (E) number of animals positive for metastases in the lungs, liver, brain and spleen for each experimental group ae presented. F) PDGF-AA and CXCL5 levels were determined in the serum from tumor-bearing animals (the experiment shown in panel A), receiving DMSO (control) or IVMT-Rx-4 (n = 6 ± SEM; ** p < 0.01, ***p < .001).

Data shown in Fig. 2 and Fig. 3 suggested that IVMT-Rx-4 could reduce *in vivo* bone metastasis, and both PDGF-AA and CXCL5 are key contributors to PC advancement of this process. An experimental study on bone metastases was conducted to examine this possibility. Fourteen days post-intracardiac injection, nearly 70% (4 out of 6) of the control group animals exhibited bone metastases, but the IVMT-Rx-4 treated group appeared metastasis free in bone (0 out of 6) (Fig. 4A and Fig. 4B). Animals were euthanized, and ex-vivo imaging of numerous organs (Fig. 4C and Fig. 4D) in bones and other organs (e.g., lungs, liver, brain and spleen, respectively) was conducted to determine if the effect seen in bone metastasis extended to metastasis in other organs. IVMT-Rx-4 partially prevented metastasis to the liver, brain and spleen, as well as to the bones (Fig. 4C, Fig. 4D, and Fig. 4E). In contrast, metastases to the lungs were not inhibited by IVMT-Rx-4 (Fig. 4D, and Fig. 4E). The reason for lack of an effect on lung metastases in this experiment is not known. The intriguing finding is that the regression of bone metastases correlated with reduced expression of two downstream effectors, PDGF-AA and CXCL5, in the serum of mice (Fig. 4F). This study indicates the prognostic importance of these two molecules for the non-invasive monitoring of IVMT-Rx-4's responses.

**Fig. 5.**
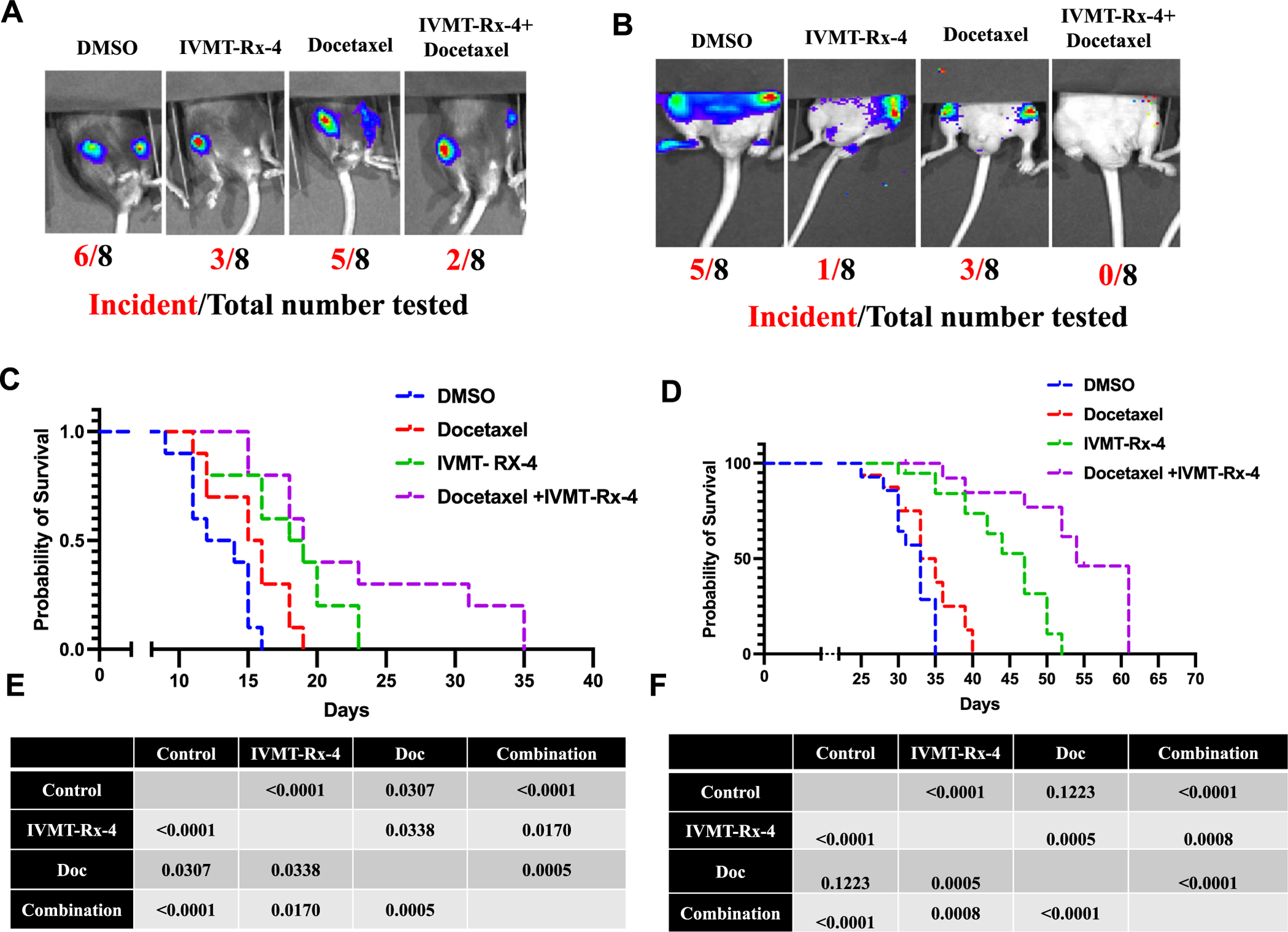
IVMT-Rx-4 in combination with docetaxel enhances survival. A and B) RM1-BM-*Luc* (A) or PC3-ML*-Luc* (B) cells were implanted I.C. in C57BL/6 and athymic nude mice, respectively. Mice received IVMT-Rx-4 (30 mg/kg body weight) and Docetaxel (5 mg/kg body weight) alone or in combination 3X in a week for a two-week period. BLI images were used to detect bone metastases (the percentage incidence of bone metastases in animals was calculated at a single time point, day 14). Values are presented at the bottom panel of the respective group (red, number of animals positive for metastasis and black, total number of experimental animals). C and D) Animals were kept until mandated euthanasia based on IACUC guidelines, considered as disease associated death. The Kaplan–Meier survival curves were generated using GraphPad in both models as described in (A) and (B), respectively. E and F) Log-rank test was performed and values between different experimental groups are presented.

We demonstrated previously that inhibition of MDA-9/Syntenin expression and associated signaling either genetically (adenovirus expressing sh*mda-9/Syntenin*) or pharmacologically (PDZ1i) enhances sensitivity of PC-stem cell xenografts to docetaxel [27], a well-established first-line therapeutic option for treating PC. Accordingly, we evaluated the potential of IVMT-Rx-4 to serve as a chemo-sensitizing compound. Our preliminary results indicated that docetaxel and IVMT-Rx-4, but not second-generation androgen deprivation therapies (ADT) (e.g., enzalutamide, abiraterone), reduced tumor cell growth in hormone-independent tumor cells (Supplementary Fig. 5 A). Indeed, IVMT-Rx-4 and abiraterone as well as docetaxel show an additive effect in hormone-dependent cell lines (Supplementary Fig. 5B). However, since our focus was on developing therapies for advanced PC, where tumor cells have become hormone-independent or have already received first-line treatment such as androgen deprivation therapy, our primary focus was on docetaxel. In hormone-independent PC, the only recommended alternative is docetaxel, either by itself or in combination with other therapeutic approaches. Therefore, combining IVMT-Rx-4 and docetaxel would currently be preferable over the other options. Both therapeutics independently suppressed metastatic incidence (measured at a single time point) and this suppression was substantially higher when animals received both regimens in combination (Fig. 5A and Fig. 5B for RM1-BM-*Luc* and PC3-ML-*Luc* cells injected animals, respectively). End point analysis (i.e., death) revealed a similar outcome, confirming that the combination therapy could robustly enhance survival as compared with control animals or animals administrated either IVMT-Rx-4 or Docetaxel alone, in both syngeneic (Fig. 5C and Fig. 5E) and nude mice (Fig. 5D and Fig. 5F). Probability of metastasis in each treatment group shows clear support of a trend towards best outcome in the combination group for both the syngeneic and athymic mice group. Estimated percent of metastasis and the 95% confidence interval is reported in Supplementary Table S7.

**Fig. 6.**
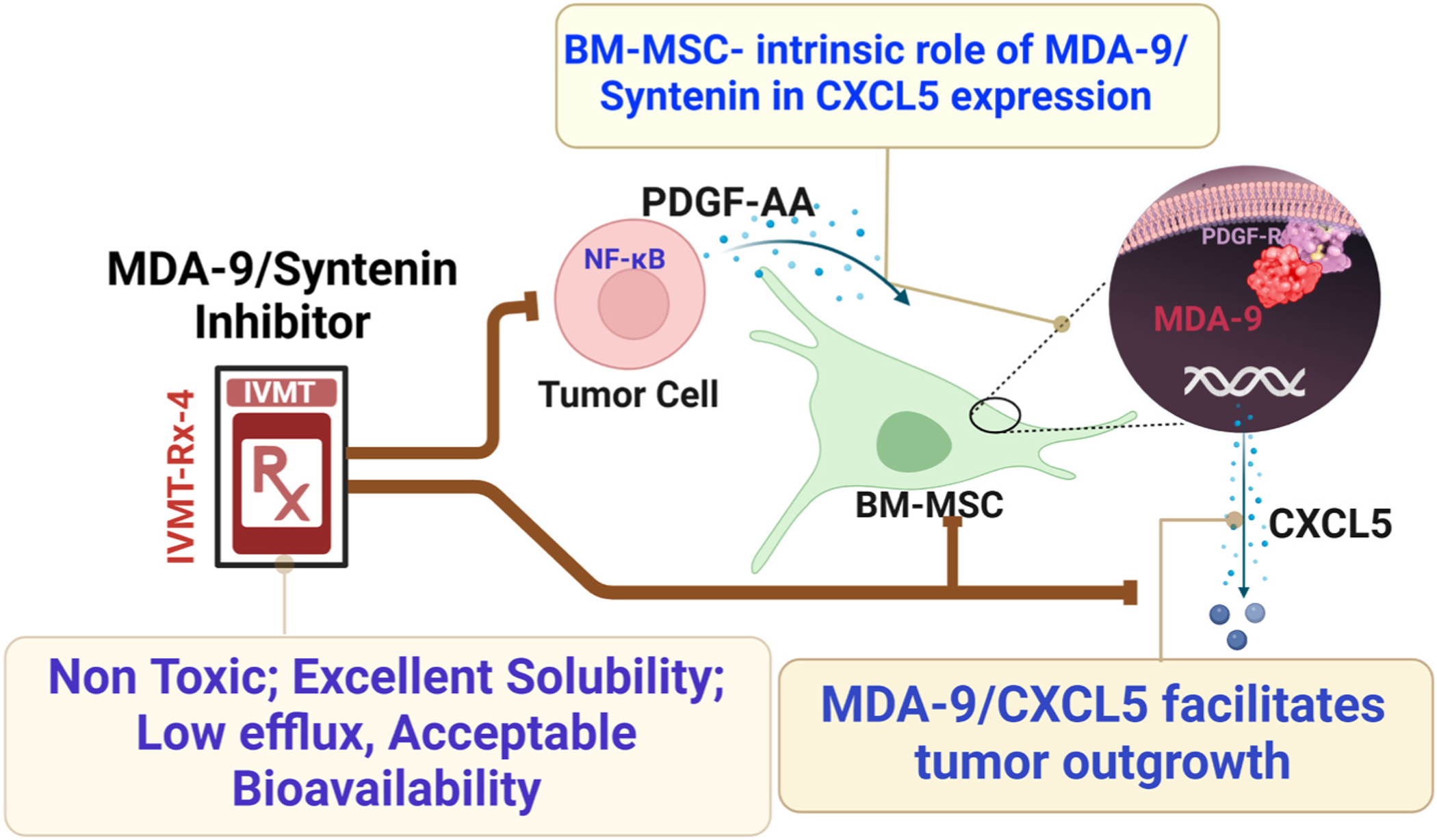
IVMT-Rx-4 inhibits PC bone metastasis through regulation of MDA-9/Syntenin activity. Our prior study established the expression of MDA-9/Syntenin in BM-MSCs provides a favorable microenvironment for invading tumor cells by secreting the chemokine CXCL5. The current study documented this pathway can be targeted using a novel MDA-9/Syntenin antagonist, IVMT-Rx-4 which is superior to PDZ1i in terms of “drug-like” properties, based on excellent solubility, higher systemic clearance and very low efflux in Caco-2 cells. The likely mode of action of IVMT-Rx-4 (in both tumor and stromal cells) is schematically presented. Created with BioRender.com.

Overall, our experiments confirm that the combination of IVMT-Rx-4 and docetaxel represent exceptional drugs-of-choice to effectively block the invasion and migration of PC cells and simultaneously negatively impact tumor growth (including an additive combination effect of IVMT-Rx-4 plus docetaxel on tumor cell growth), thereby suppressing PC bone metastasis and improving therapeutic outcomes (Fig. 6).

## 4. Discussion

Treating advanced metastatic PC poses significant clinical challenges. It mandates the use of innovative techniques to identify possible therapeutic targets that can be effectively nullified with medications to yield positive outcomes in patients. Due to the high mortality rate of PC bone metastases and the eventual ineffectiveness of traditional treatments, it is imperative to adopt new targets and identify effective therapeutics focused on these targets. MDA-9/Syntenin is such a target that has been shown to be highly expressed in various types of cancer, including metastatic PC [9,17]. Genetic and pharmacological approaches in multiple model cancer systems indicate that MDA-9/Syntenin is indeed a promising molecular target mediating cancer aggression and metastasis [15,17,24,27,28]. Our current work focuses on a novel MDA-9/Syntenin-antagonist, IVMT-Rx-4, which effectively inhibits invasion and PC-bone metastatic progression (as summarized in Fig. 6) through interrupting the signaling of two critical proteins, PDGF-AA and CXCL5, expressed by tumor and bone marrow stromal cells, respectively.

The binding interactions of IVMT-Rx-4 with the PDZ1 pocket reveal key structural insights that highlight its potential as an effective inhibitor. One aromatic group of IVMT-Rx-4 occupies the deeper hydrophobic region of the binding cavity, while the second aromatic moiety engages one of three shallower hydrophobic regions. Charged interactions involving the conjugated oxygen and nitrogen play a critical role in stabilizing favorable binding poses. Notably, the less shallow hydrophobic pocket at the bottom of the binding cavity accommodates the bulkier aromatic moiety, but this configuration weakens the charged interaction with the conjugated oxygen, resulting in higher free-energy poses. These findings suggest that IVMT-Rx-4 consistently leverages a balance of hydrophobic and charged interactions to effectively fill the binding pocket, providing a structural basis for its potential as a PDZ1 inhibitor and a promising avenue for therapeutic exploration.

Despite our primary data from Fig. 2D (which shows that IVMT-Rx-4 has no effect on *mda-9/Syntenin* manipulated tumor cells) and Fig. 3E (indicating that IVMT-Rx-4 is inactive in promoting CXCL5 expression in BMSCs isolated from *mda-9/Syntenin* null animals), this data does not rule out any potential off-target effects, especially since we observed significant efficacy with only marginal binding affinity. It is important to note that although the shift was marginal, it appears the interruption was retained for at least 30 mins, which may cause sufficient interruption of MDA-9/Syntenin downstream signaling. However, it is also possible that IVMT-Rx-4 displays “rebinding” activity (bind, dissociate, and rebind), thereby prolonging target occupancy [29,30], which needs further experimental validation. The current study is primarily focused on a specific target interaction rather than exploring binding in a broader context, such as the effects of IVMT-Rx-4 on other PDZ domain-containing proteins. While the potential binding of IVMT-Rx-4 to the first domain of PDZ has been computationally demonstrated, it has not yet been experimentally validated, nor have the effects of this domain on its potential partners been explored (notably, MDA-9/Syntenin interacts with numerous proteins and may form multimeric complexes, including MDA-9/MDA-9 interactions) [10]. We are currently examining the preferential binding of MDA-9/Syntenin with its companion protein(s) and the potential effects of IVMT-Rx-4 through co-precipitation pull-down assays.

PDGFs are potent stimulants for mesenchymal stromal cells and significantly impact various crucial biological processes, including tissue remodeling, wound healing, metastasis, and tumor survival [31,32]. PDGFs can be produced by both malignant and non-malignant cells, such as endothelial cells and macrophages [33,34]. Furthermore, tumor cells and stromal cells, including blood vessels, fibroblasts, and myofibroblasts, express PDGFR (α or β) receptors [35] that can promote paracrine production and secretion. Accordingly, blocking PDGF expression should provide an effective means of controlling disease. Within the tumor microenvironment, the tumor releases PDGF-AA, which draws in MSCs and triggers the BMP-Smad1/5/8 pathway, initiating osteogenic differentiation [36,37]. Unfortunately, therapeutic targeting of PDGF has demonstrated little efficacy in clinical contexts. According to our previous findings, MDA-9/Syntenin controls the release of the growth factor PDGF-AA from tumor cells by activating the NF-κB pathway. It also directly stimulates BMSCs to increase production of the chemokine CXCL5 through regulation of the Hippo-signaling pathway in an MDA-9/Syntenin-dependent manner [4]. It is noteworthy that, another member of this gene family, PDGF-BB, has been shown to regulate the Hippo pathway in pancreatic stellate cells [38] as well as in pancreatic cancer [39]. The current work demonstrates that the new MDA-9/Syntenin antagonist IVMT-Rx-4 effectively inhibits the activation of NF-κB, leading to a reduction in the production of PDGF-AA from tumor cells.

CXCL5 plays a vital role in both attracting tumor cells towards specific locations (chemotaxis) and creating a favorable environment that promotes the growth of neoplastic cells [40,41]. In advanced PC, inhibiting CXCR2, the receptor for CXCL5, decreases aggressiveness and induces senescence by facilitating the invasion of tumor-associated macrophages [42,43]. Previous research has shown that CXCL5 plays a crucial role in the spread of both PC and breast cancer to bones. In our earlier study, we discovered that MDA-9/Syntenin/CXCL5 contributes to bone metastasis in PC [4]. PDGF-AA activates the Hippo signaling pathway, which in turn regulates the production of CXCL5 from BMSCs. This process is dependent on the presence of MDA-9/Syntenin. The application of IVMT-Rx-4 to suppress MDA-9/Syntenin provides significant benefits in reducing bone metastases by controlling the release of CXCL5 from BMSCs.

Like other PDZ domain containing proteins, targeting MDA-9/Syntenin is challenging. Several approaches, including small molecule- or peptide-based, have been developed and validated both *in vitro* and *in vivo* [13]. Although most showed reasonable activity, the major concerns that remained involved weak affinities, particularly for small molecules. In a recent study, we developed and evaluated a bivalent molecule consisting of both a peptide and a small molecule (IVMT-Rx-3), which demonstrated substantial biological efficacy without strong affinity for its target, MDA-9/Syntenin [24]. Although we did not compare multiple MDA-9/Syntenin compounds in any specific setting, we anticipate that each compound may exhibit varied efficacy based on factors such as target affinity, domain specificity, and other pharmacokinetic and pharmacodynamic characteristics (recently reviewed by us [13]). The current antagonist, IVMT-Rx-4, is not an exception and displays low binding affinity for MDA-9/Syntenin. However, the efficacy data remains encouraging and demonstrates druggable ADME properties without provoking any visible toxicity in animals. Further studies are required to accept or rule out the possibility of consecutive binding, rebinding or long-lasting binding (partially shown in Fig. 1B). There is also accumulating evidence suggesting that long lasting target binding of therapies positively influences *in vivo* pharmacokinetic properties and bio-efficacy [30]. The pharmacokinetic profile demonstrates rapid systemic absorption across all non-intravenous routes, with T_max_ values within 0.5 h, indicating efficient uptake (Supplementary Fig. S4). Notably, oral administration achieved a relatively high C_max_ compared to intravenous dosing, suggesting favorable oral bioavailability and warranting further exploration of the oral route [44]. Extended plasma half-life indicates absorption-rate-limited kinetics [45]. These findings support the potential of multiple non-invasive routes, including oral delivery, to achieve therapeutically relevant systemic exposure of IVMT-Rx-4.

Although metastasis is the most common cause of cancer-associated death from solid tumors, the current drug discovery pipelines predominantly focus on targeting primary tumors. Despite numerous scientific challenges that can be attributed to the existence of numerous overlapping and compensating pathways and inadequate understanding and specificity of signaling pathway blockers, a variety of anti-metastatic drugs have been validated in pre-clinical settings, and only some of these have been tested in the clinic. Unfortunately, to date no-anti-metastatic drug, used as a single therapy, has provided promising results in the clinic. MMP inhibitor(s) failed to show any promise in clinical studies due to overlapping functions of other proteases. Actin-cytoskeleton targeting medicines were highly toxic due to non-specificity. Additionally, integrin targeting medicines such as cilengitide, even in conjunction with temozolamide, did not increase survival in GBM compared with historical treatments [46]. Late-stage failures of these different classes of medicines diminish research agendas to define anti-metastasis drugs and evaluate them in the clinical setting, both in the pharmaceutical and academic arenas.

Developing a clinical trial is often not prudent unless a drug exhibits no or minimal harmful effect(s) as an endpoint when using therapeutically efficacious dosing. Thus, a combination of a drug with anti-metastatic activity (with limited toxicity) and a validated and well tested (and tolerated) cytotoxic chemotherapeutic agent might provide a path forward with a higher likelihood of successful therapeutic outcomes. Docetaxel is a widely accepted and effective treatment for PC that is often used as the first-line therapy [47,48]. The involvement of MDA-9/Syntenin in chemoresistance has been previously documented, rationalizing our evaluation of IVMT-Rx-4 as a chemosensitizer in PC. As predicted, IVMT-Rx-4 increased the sensitivity of PC cells to docetaxel *in vitro*. Furthermore, IVMT-Rx-4 treatment, either alone or in combination with docetaxel, demonstrated a substantial decrease in the progression of PC to the bones and other organs *in vivo* in animal models. Further investigations are necessary to elucidate the precise molecular mechanism underlying this enhanced effect. Additionally, our earlier studies on PC stem cells confirmed regulatory functions of MDA-9/Syntenin in survival, stemness, and chemoresistance [27]. In a xenograft study, targeting MDA-9/Syntenin with genetic or pharmacological approaches increased sensitivity to docetaxel. A comparable role of MDA-9/Syntenin has been documented by Iwamoto *et al.* in colorectal cancer stemness and resistance to oxaliplatin [49]. MDA-9/Syntenin contributes to cisplatin resistance in head and neck cancer stem cells, and its genetic reduction can reinstate drug sensitivity [50]. Although we did not investigate the mechanism in this study, experimental evidence confirms improved survival when IVMT-Rx-4 and docetaxel are used in combination in a metastatic PC bone metastasis model. Considering the positive role of MDA-9/Syntenin in promoting bone metastasis by controlling both intrinsic and extrinsic mechanisms in PC tumor cells, we believe that IVMT-Rx-4 is a promising and potentially significant pharmacological inhibitor for PC. With additional toxicological studies- in particular, the impact on bone resident cells and bone homeostasis and further optimization in dosing, formulation and route of administration-IVMT-Rx-4 has the potential to become an effective drug for preventing PC tumor cell invasion and bone metastasis.

## Supplementary Material

MMC5

MMC4

MMC1

MMC9

MMC3

MMC8

MMC2

MMC13

MMC7

MMC12

MMC10

MMC6

MMC11

## Figures and Tables

**Table 1:** Solubility and Permeability of IVMT-Rx-4 vs. PDZ1i.

Assay	IVMT-Rx-4	PDZ1i
Thermodynamic solubility in PBS, pH 7.4	~160	10
Log D by shake flask (pH 7.4)	1.49	0.5
Caco-2 Bidirectional	A-B: 21.5 × 10^−6^ cm/s B-A: 19.9 × 10^−6^ cm/s Efflux 0.92	A-B: 0.04 × 10^−6^ cm/s B-A: 11.5 × 10^−6^ cm/s Efflux 300

**Table 2:** ADME Assays IVMT-Rx-4 vs. PDZ1i.

Assay	IVMT-Rx-4	PDZ1i
Stability in liver microsomes (human, monkey, dog, rat, mouse) with both NADPH and UDPGA added	Minimal degradation: > 3.5 hr t½ in all species	Minimal degradation: > 4 hr t½ in all species
Drug-substrate based CYP Inhibition (1A2, 2A6, 2B6, 2C8, 2C9, 2C19, 2D6, 2E1, 3A4_M, 3A4_T)	2E1 (~8 μM); 3A4 (~10 μM), minimal (>10 μM) with others	2C8 (~2 μM); > 2E1 (~8 μM), minimal (>10 μM) with others
Time Dependent Inhibition (TDI), CYP4A only	No TDI	No TDI
PXR assay	No induction	No induction
hERG assay by manual patch-clamp system (n = 3)	> 30 mM	> 30 mM
Mini-Ames Assay (2-strain option: TA98 & TA100)	Weakly positive for 2 strains	Negative

## Data Availability

Data will be made available on request.

## Appendix A. Supporting information

Supplementary data associated with this article can be found in the online version at doi:10.1016/j.phrs.2026.108164.
