# Supplementary figures and images for: MDA-9/Syntenin small molecule inhibitor IVMT-Rx-4 blocks prostate cancer bone metastasis
Source: Pharmacol Res. Author manuscript; Available in PMC 2026 Jul 25. (PMC13401508; doi:10.1016/j.phrs.2026.108164)

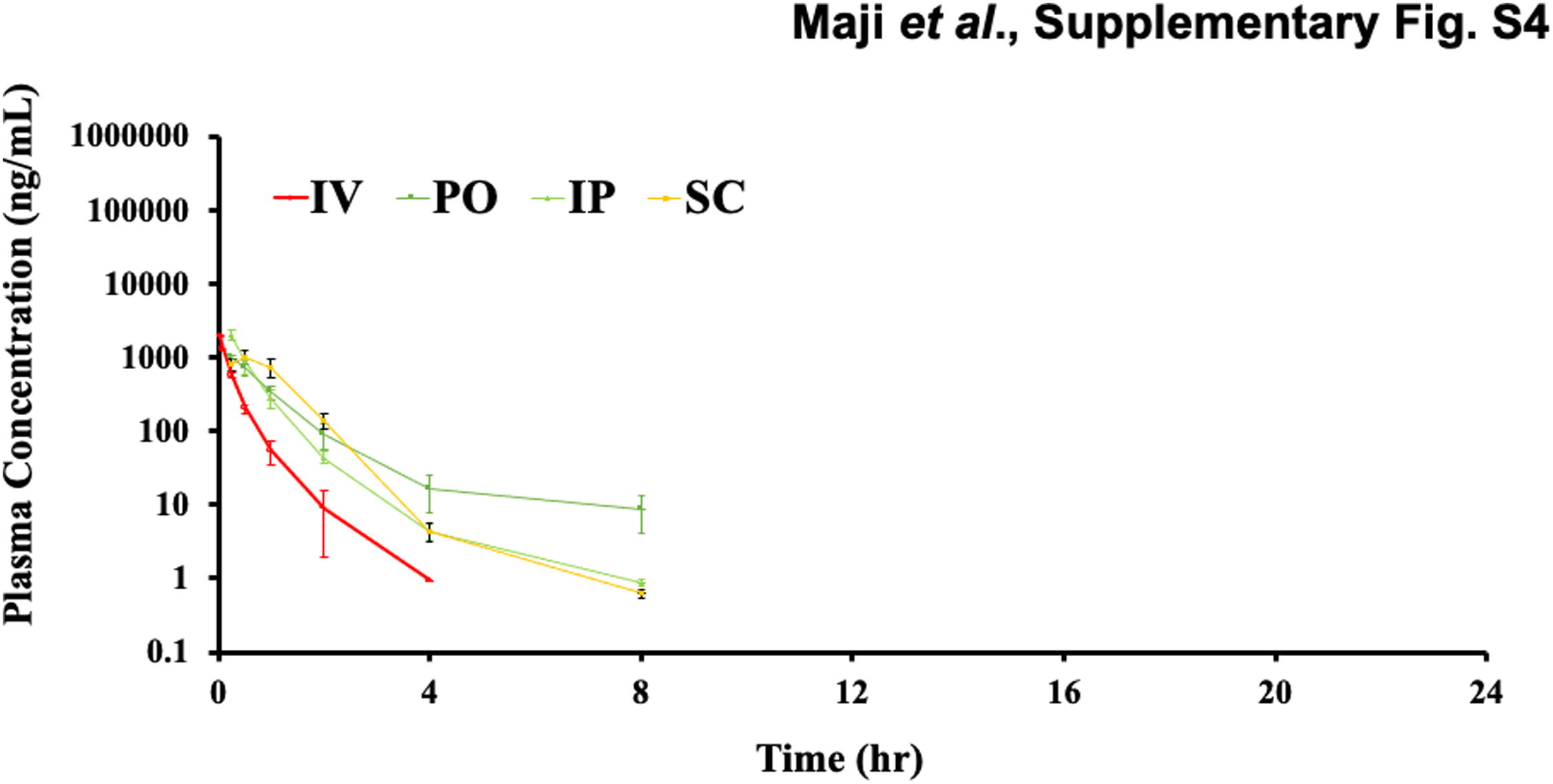

Supplement: MMC5 [file NIHMS2174343-supplement-MMC5.jpg]

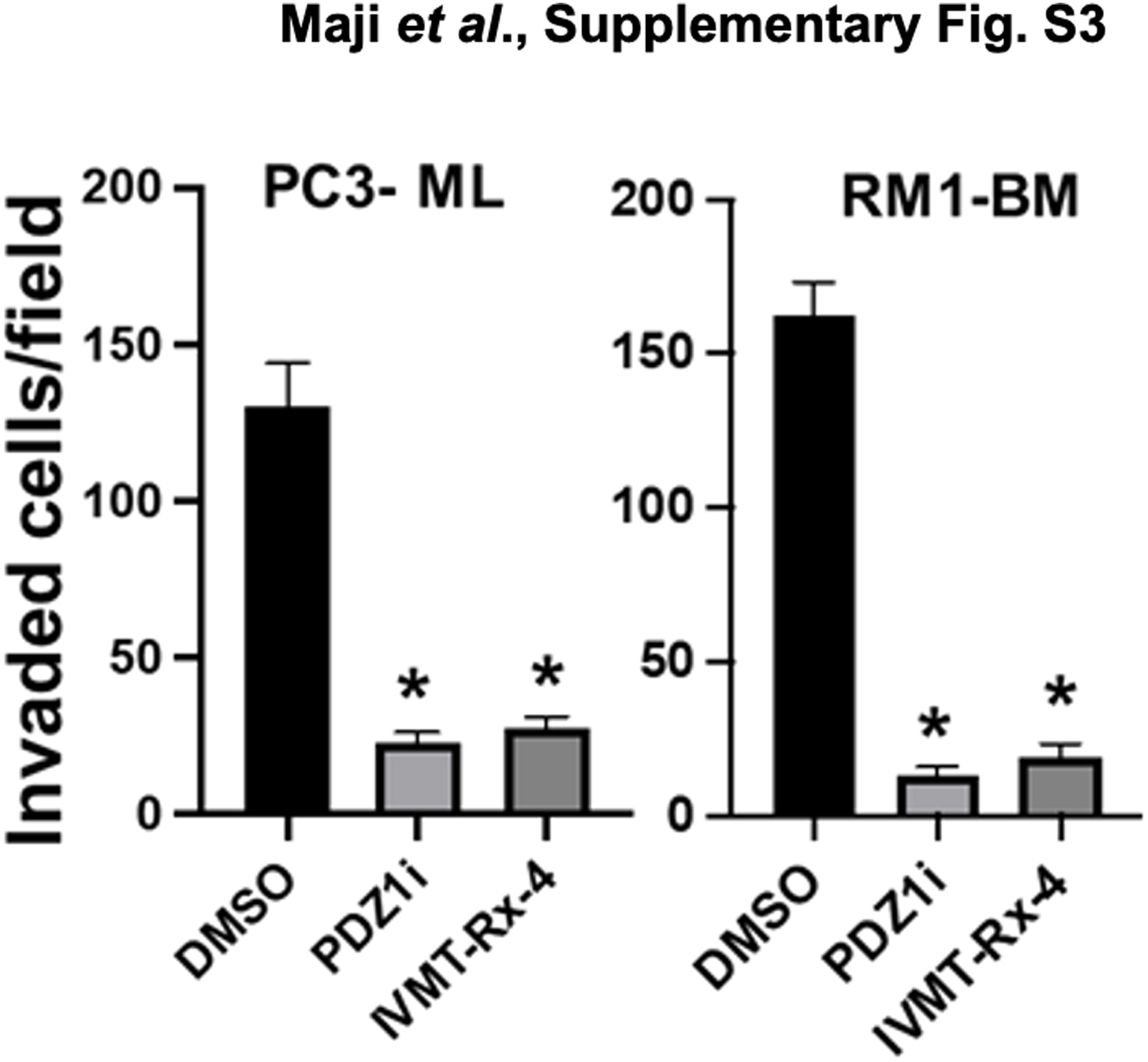

Supplement: MMC4 [file NIHMS2174343-supplement-MMC4.jpg]

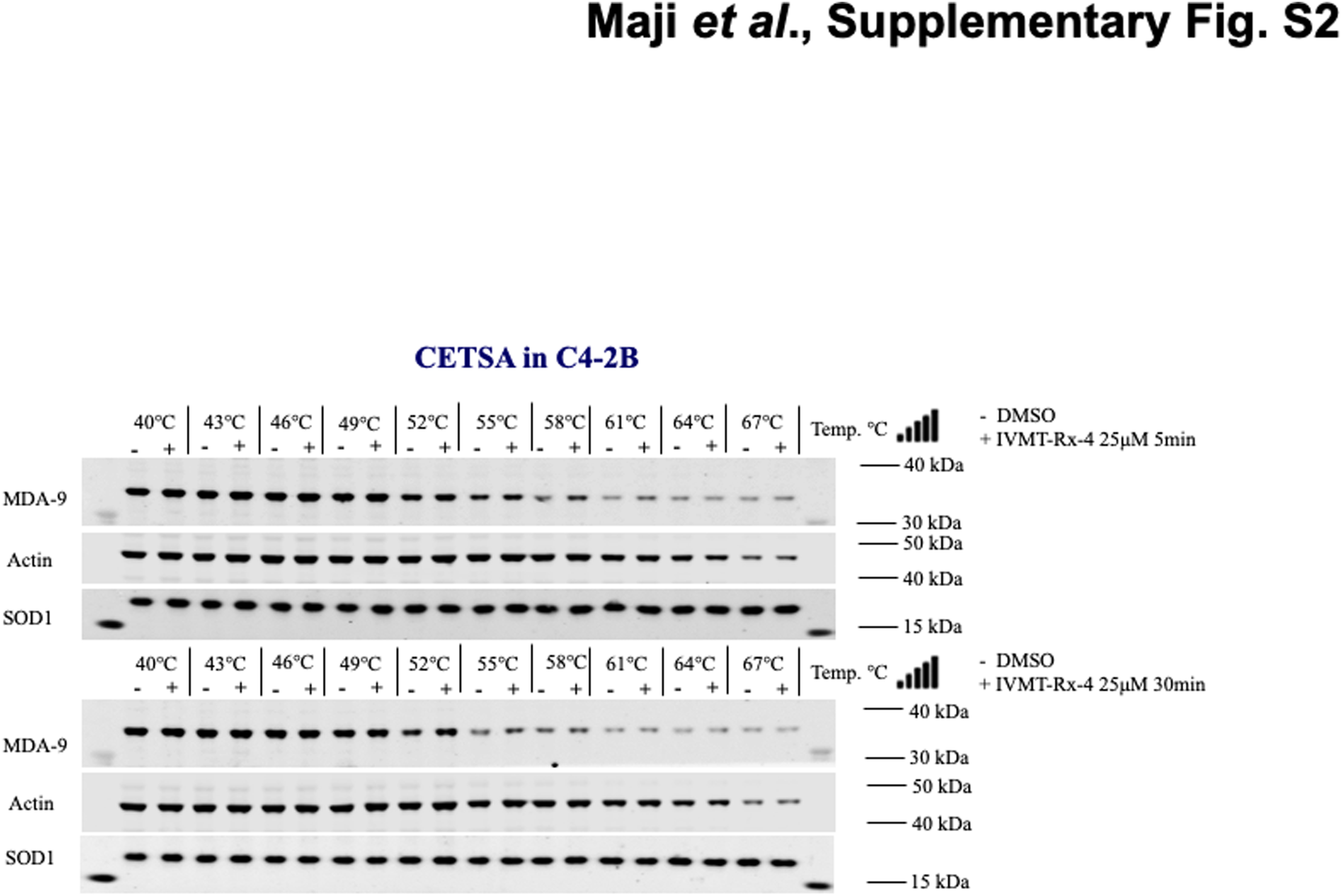

Supplement: MMC3 [file NIHMS2174343-supplement-MMC3.jpg]

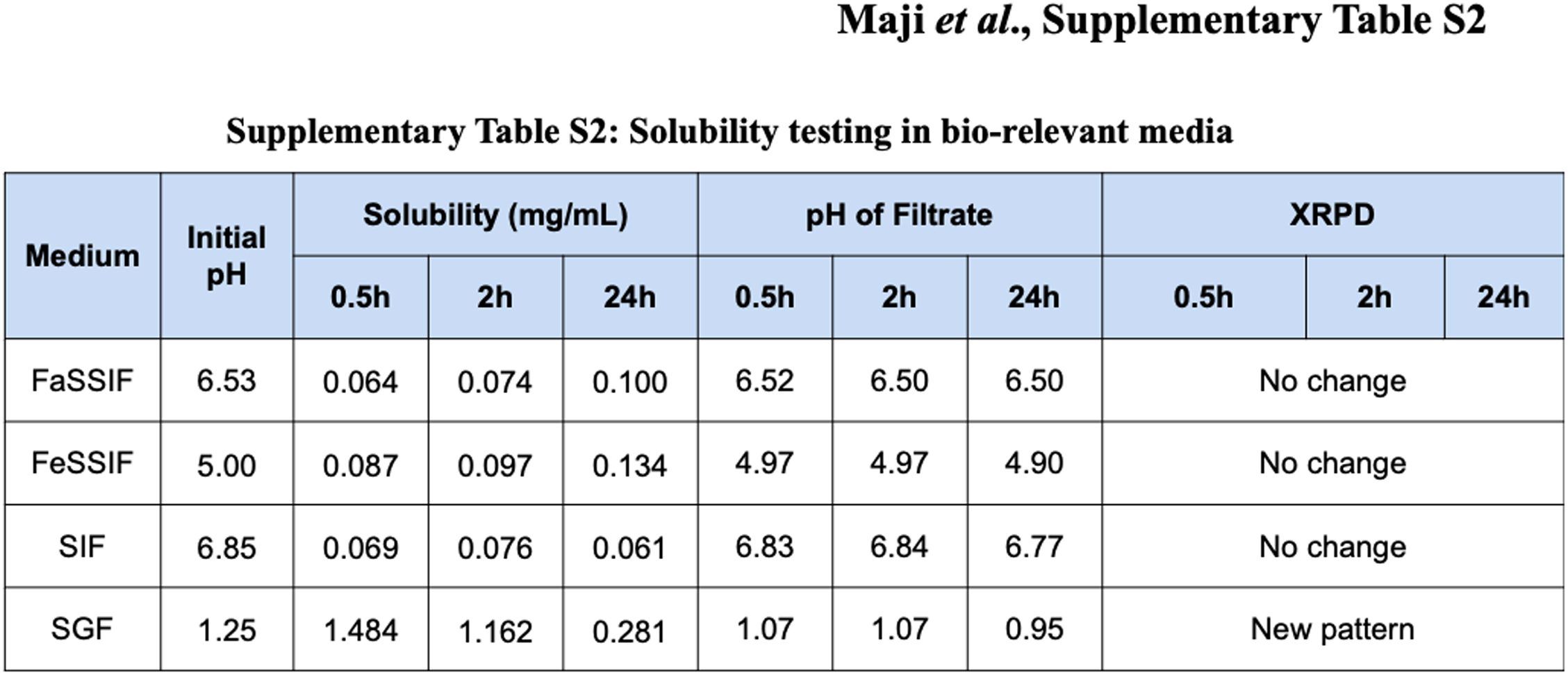

Supplement: MMC8 [file NIHMS2174343-supplement-MMC8.jpg]

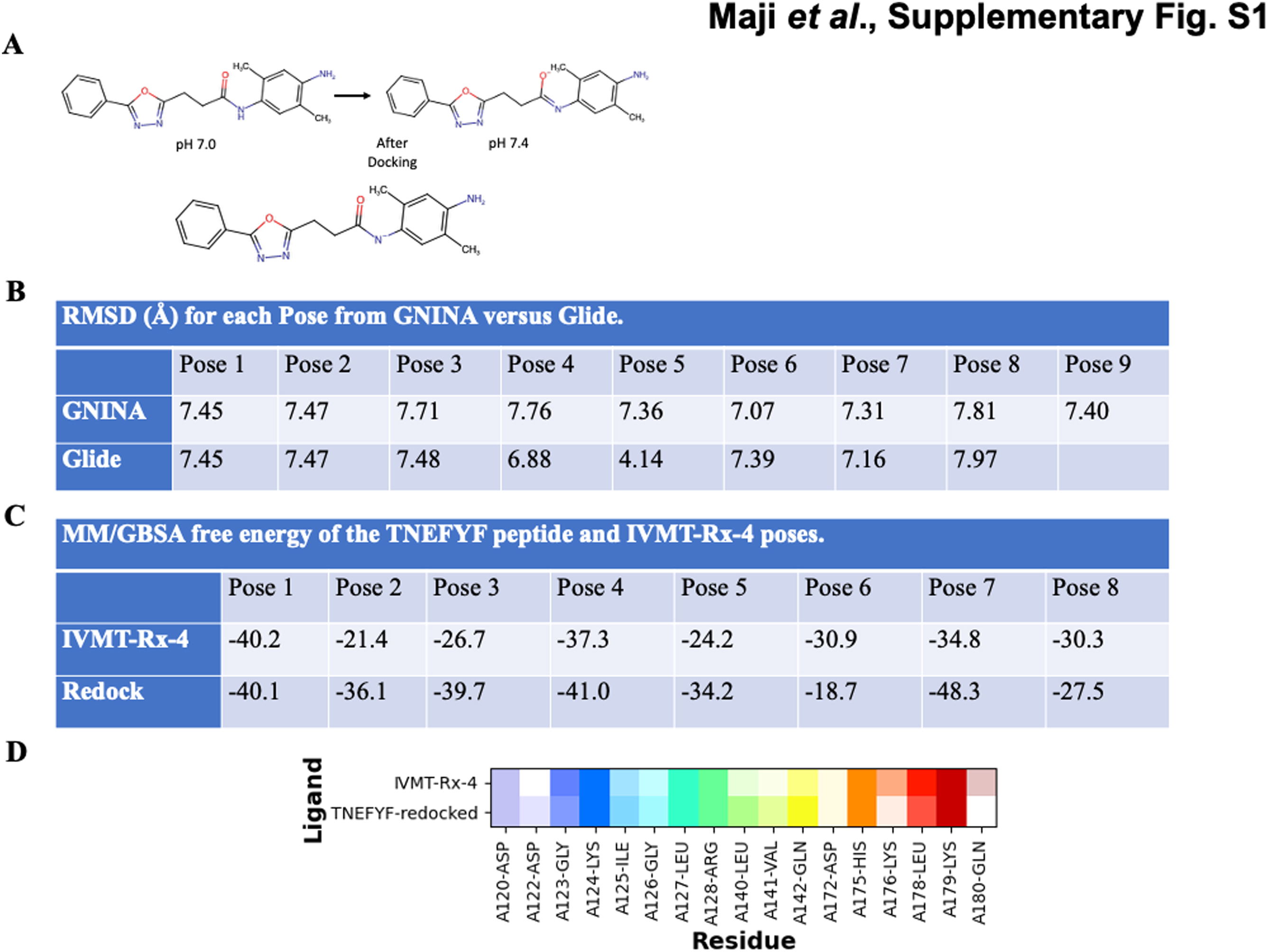

Supplement: MMC2 [file NIHMS2174343-supplement-MMC2.jpg]

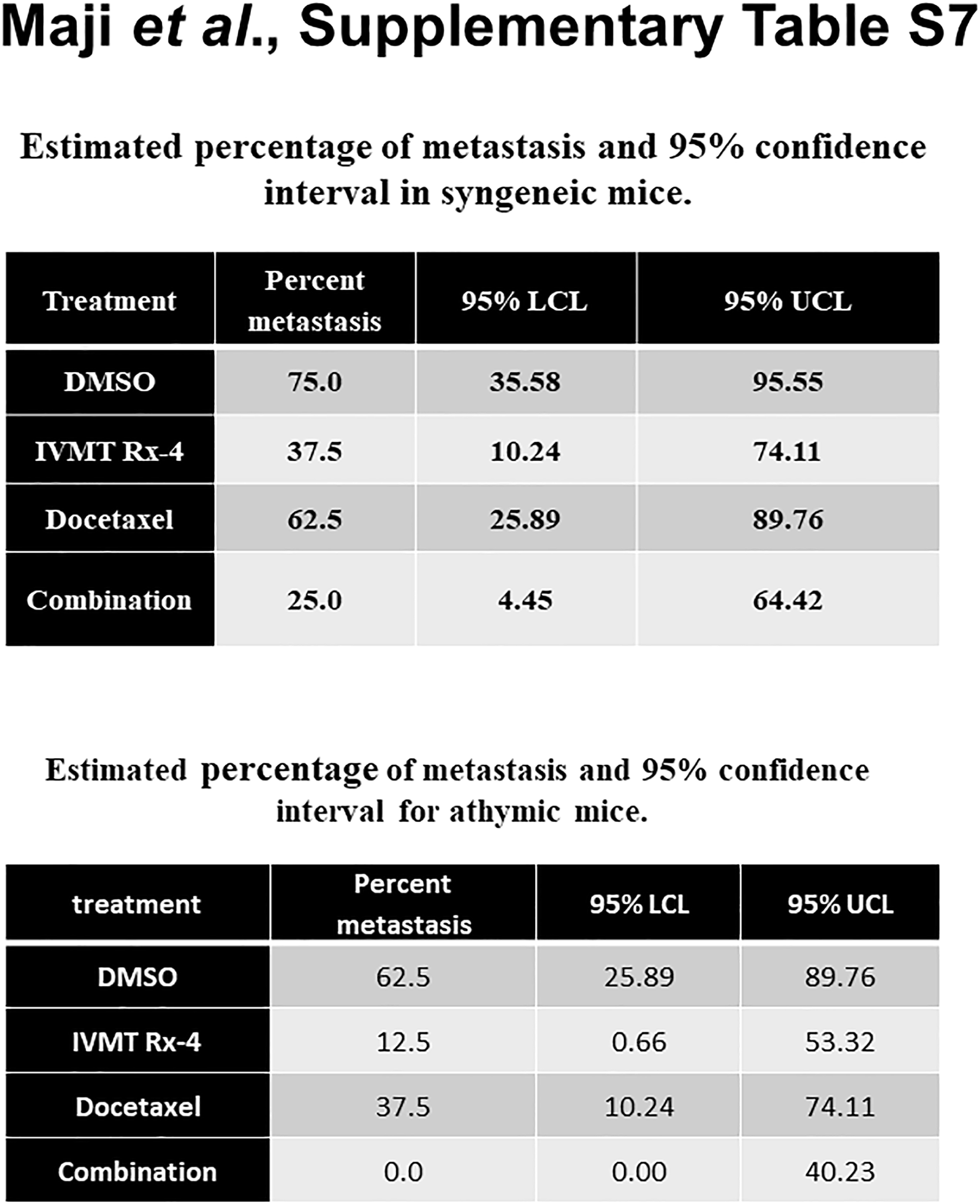

Supplement: MMC13 [file NIHMS2174343-supplement-MMC13.jpg]

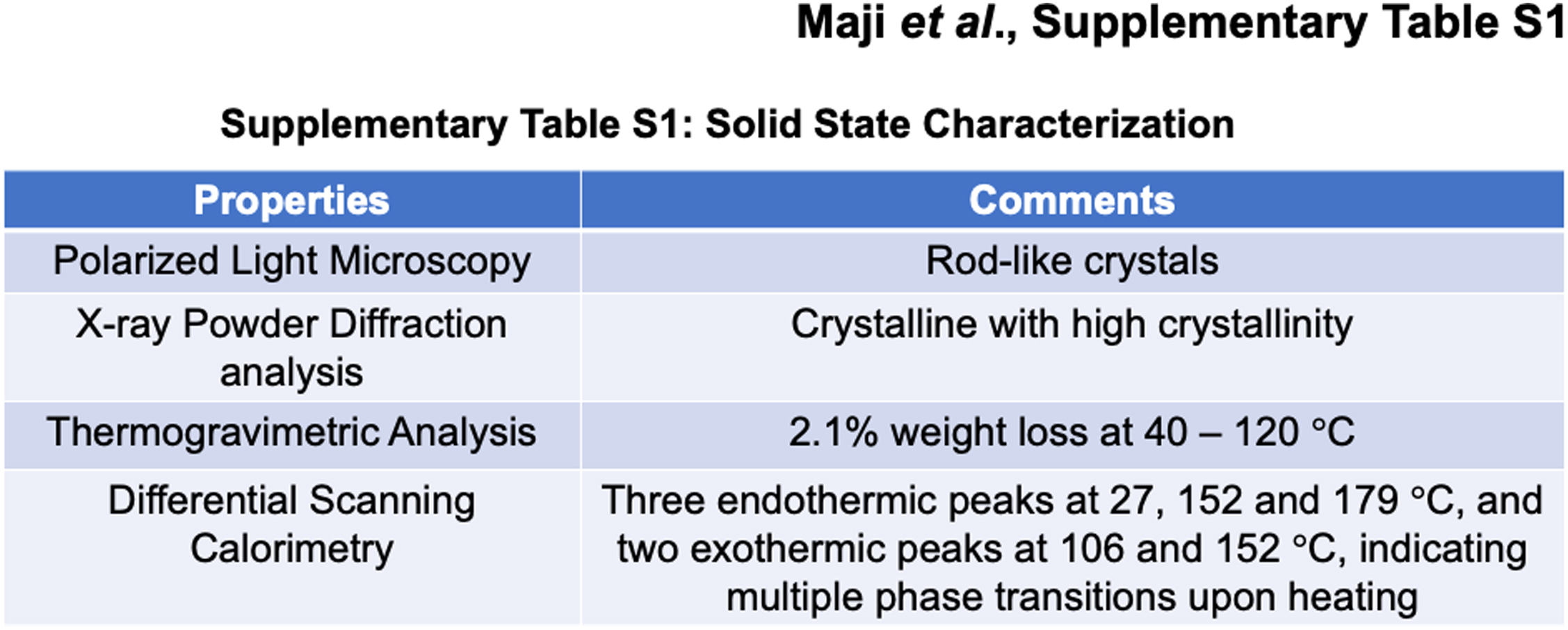

Supplement: MMC7 [file NIHMS2174343-supplement-MMC7.jpg]

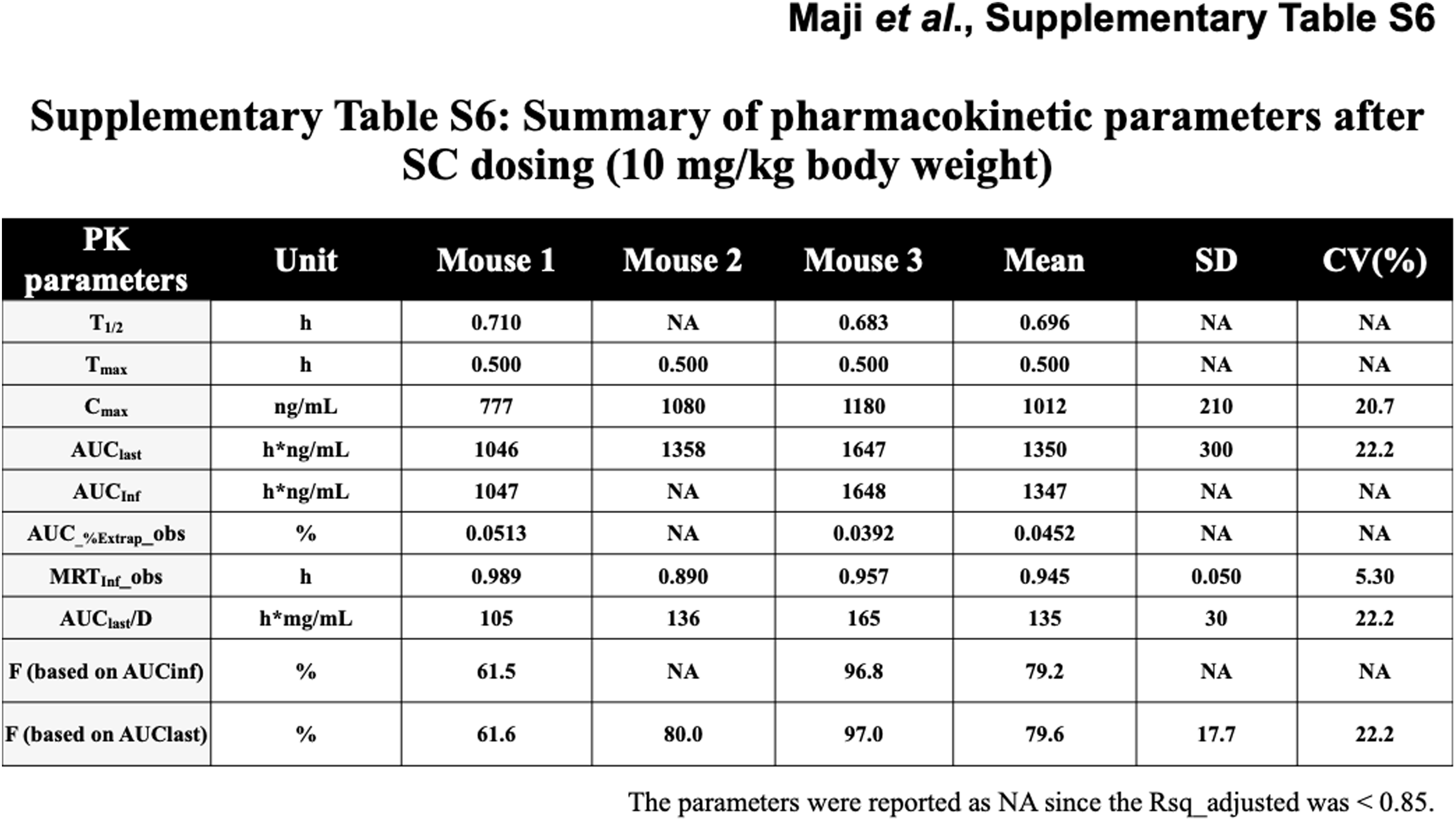

Supplement: MMC12 [file NIHMS2174343-supplement-MMC12.jpg]

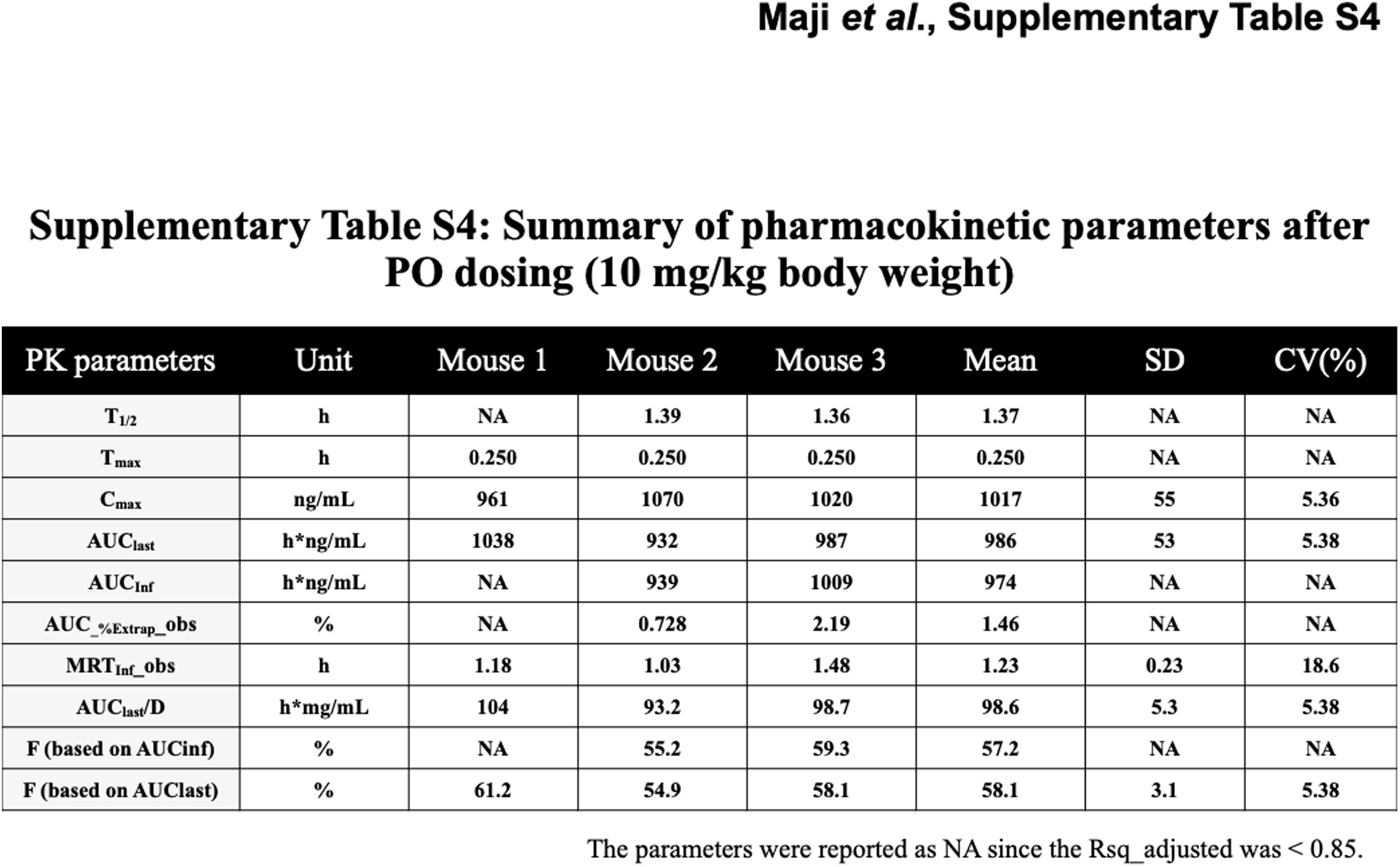

Supplement: MMC10 [file NIHMS2174343-supplement-MMC10.jpg]

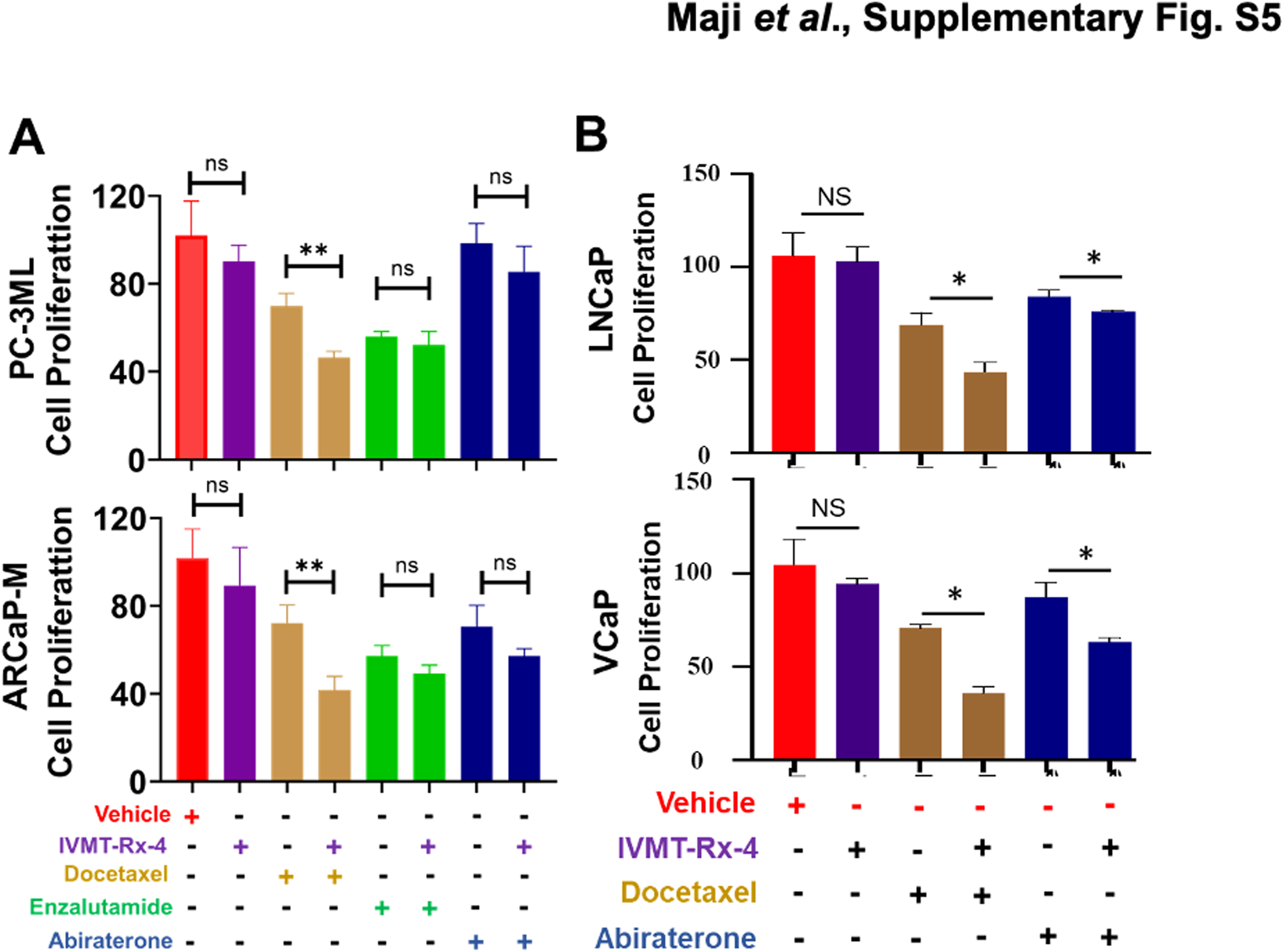

Supplement: MMC6 [file NIHMS2174343-supplement-MMC6.jpg]

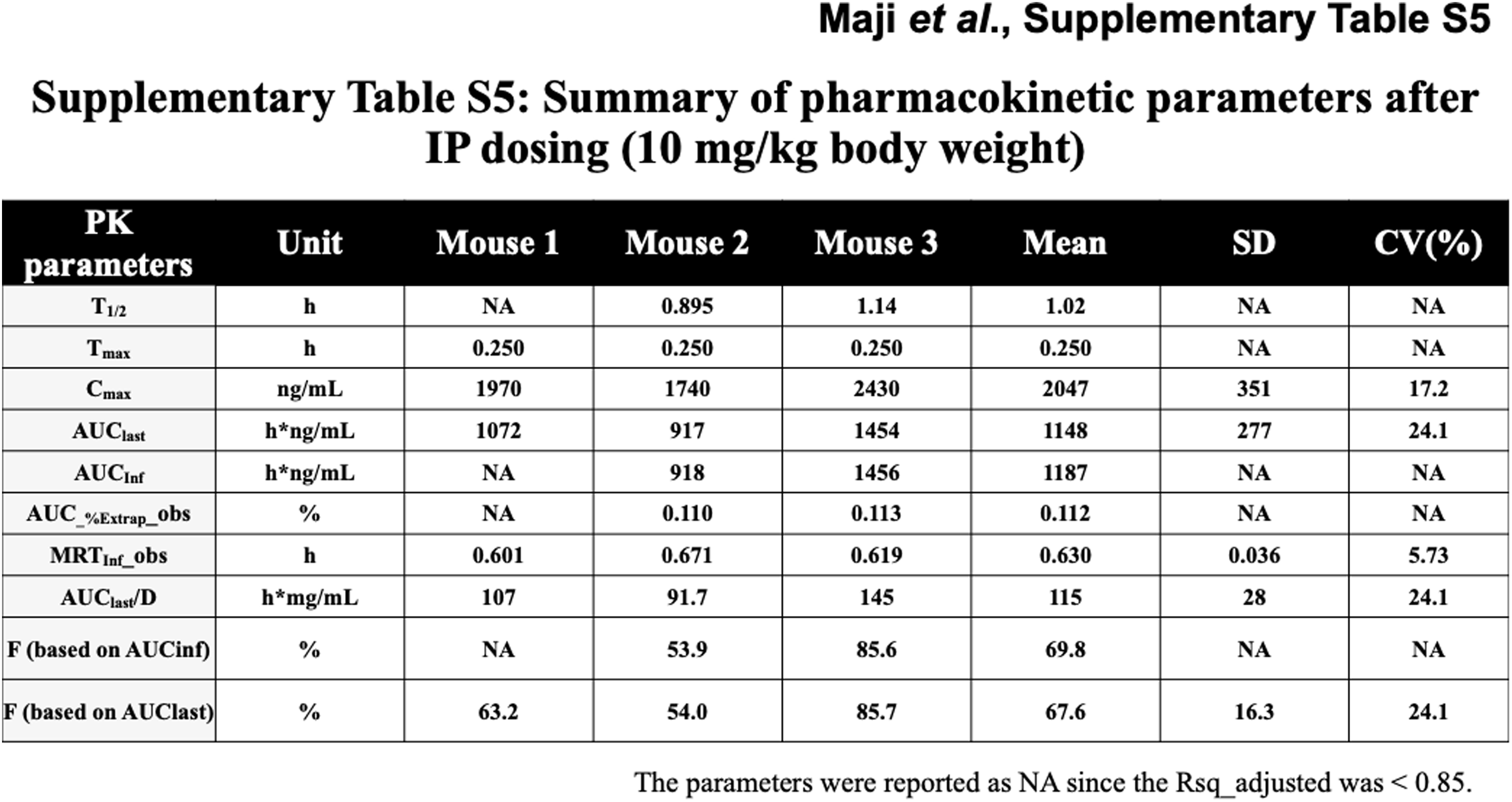

Supplement: MMC11 [file NIHMS2174343-supplement-MMC11.jpg]
