# Supplementary material for: MDA-9/Syntenin small molecule inhibitor IVMT-Rx-4 blocks prostate cancer bone metastasis
Source: Pharmacol Res. Author manuscript; Available in PMC 2026 Jul 25. (PMC13401508; doi:10.1016/j.phrs.2026.108164)
Supplement: MMC1 [file NIHMS2174343-supplement-MMC1.docx]

**Supplementary Documents_ Maji *et al***

**IVMT-Rx-4 Synthesis Scheme**

*Synthesis of compound 3:*A mixture of compound **1** (105 g, 771 mmol, 1.00 eq) and compound **2** (84.9 g, 848 mmol, 1.10 eq) in Ethyl acetate (1200 mL) was stirred at 28 °C for 10 hrs. TLC (Petroleum ether/ Ethyl acetate = 0/ 1, material Rf = 0.50, product Rf = 0.10) showed the material was consumed and a new spot was formed. LCMS (EC1383-1-P1A1) showed the desired MS was detected. The reaction mixture was filtered, and the cake was washed with Ethyl acetate (100 mL * 2) and dried to give a crude product. The crude product compound 3 (168 g, 711 mmol, 92.2% yield) as a white solid was used into the next step without further purification. [M+H]^+^ was 259.3. ^1^H NMR: EC1383-1-P1A1 (400 MHz DMSO-d6), δ 12.27-11.93 (m, 1H), 10.31 (s, 1H), 9.91 (s, 1H), 7.90-7.82 (m, 2H), 7.61-7.45 (m, 3H), 2.49-2.40 (m, 4H).

*Synthesis of compound 4:* To a solution of P_2_O_5_ (100 g, 711 mmol, 43.9 mL, 2.00 *eq*) in H_2_SO_4_ (500 mL) was added compound **3** (168 g, 711 mmol, 1.00 *eq*) under N_2,_ the mixture was stirred at 28 °C for 10 hrs. LC-MS showed Reactant 1 was consumed completely and one main peak with desired mass was detected (LC-MS, EC1383-2-P1A, RT = 0.381 min, m/z = 219.3). The reaction mixture was poured into water and ice (5000 mL) at 0 °C and precipitate was formed. The mixture was filtered, and the cake was dried to give a residue. The residue was triturated with petroleum ether (500 mL) to give compound **4** (103 g, 472 mmol, 66.4% yield) as a white solid was used into next step without further purification. [M+H]^+^ was 219.3. ^1^H NMR: EC1383-2-P1A (400 MHz DMSO*-d_6_*), *δ* 12.38 ( s, 1H), 7.98 (dd, *J* = 1.7, 7.7 Hz, 2H), 7.67-7.54 (m, 3H), 3.14 (t, *J* = 7.1 Hz, 2H), 2.81 (t, *J* = 7.1 Hz, 2H).

*Synthesis of compound 6 (or IVMT-Rx-4):* To a mixture of compound **4** (95.0 g, 435 mmol, 1.00 *eq*) and compound **5** (59.3 g, 435 mmol, 1.00 *eq*) in DMF (1200 mL) was added EDCI (100 g, 522 mmol, 1.20 *eq*), HOBt (70.6 g, 522 mmol, 1.20 *eq*) and TEA (52.9 g, 522 mmol, 72.7 mL, 1.20 *eq*) in one portion under N_2_. The mixture was stirred at 20 °C for 5 hrs. LC-MS showed Reactant 1 was consumed completely and one main peak with desired m/z was detected (LC-MS, EC1383-4-P1A, RT = 0.346 min, m/z = 337.3). The reaction mixture was added water (3000 mL), then filtered and concentrated under reduced pressure to give a residue. The cake was what we need. The crude product was triturated with solvent (800 mL, Petroleum ether/ Ethyl acetate = 1/ 4) at 20 °C for 20 min. Compound 6 or IVMT-Rx-4 (100 g, 281 mmol, 64.6% yield, 94.7% purity) was obtained as a light brown solid. [M+H]^+^ was 337.3. ^1^H NMR: EC1383-4-P1A (400 MHz DMSO*-d_6_*), *δ* 9.12 (s, 1H), 8.02-7.93 (m, 2H), 7.64-7.53 (m, 3H), 6.75 (s, 1H), 6.40 (s, 1H), 4.68-4.59 (m, 2H), 3.25-3.16 (m, 2H), 2.82 (t, *J* = 7.1 Hz, 2H), 1.95 (d, *J* = 2.4 Hz, 6H).

**Methods for IVMT-Rx-4 Docking**

The TNEFYF peptide from PDB ID: 1W9E (1) crystal structure was redocked using GNINA v1.1(2) and Glide from Schrodinger Suite version 2024-4 (3). In both programs, the box was centered around the PDZ1 domain at 15x35x50. In GNINA the box size was 25x25x25 Å. In Schrodinger Maestro, several preprocessing steps were utilized for the protein and the TNEFYF peptide. The protein preparation workflow was used to assign bond orders, replace hydrogens, create disulfide bonds and generate protonation states using Epik at pH 7.4. Hydrogen-bond assignments were optimized and minimized via restrained minimization. In Schrodinger Maestro, the inner box was 20 Å and the outer box was 10 Å and docking was run with standard precision. RMSD was calculated using an in-house python script. Glide had the lowest RMSD of peptide redocking and Glide was used to dock IVMT-Rx-4 into the PDZ1 domain. IVMT-Rx-4 was prepared using LigPrep to generate possible states at pH 7.4 using Epik and generate tautomers. Interaction Fingerprints and MM/GBSA values were obtained from Canvas and Prime in Schrodinger Suite version 2024-4.

**Solid-phase characterization**

*Differential Scanning Calorimetry (DSC)*

Thermal properties of IVMT-Rx-4 were evaluated using differential scanning calorimetry on a TA instrument (Discovery DSC 250). Approximately 1.48 mg of sample was weighed into a non-hermetically sealed aluminum pan and scanned at a heating rate of 10 ºC/min over a temperature range of RT to 300 °C. Heat flow was plotted against temperature using TRIOS software to create the curve used for analysis.

*Dynamic Vapor Sorption (DVS)*

Hygroscopicity and moisture absorption of IVMT-Rx-4 were assessed using dynamic vapor sorption on a DVS instrument (SMS, DVS Intrinsic). Samples 20.4 mg in size were first treated at 25°C for several hours while under nitrogen flow. They were then treated with water vapor at various partial pressures with relative humidities which ranged from 0 to 90% at 25°C.

*Particle Size Distribution (PSD)*

PSD of the IVMT-Rx-4 was determined by Malvern Mastersizer 3000 using wet method. About 15 mg of sample was pre-dispersed using 1 mL of water. After mixing completely, PSD was analyzed with the parameters in Table below.

| Instrument | Malvern Mastersizer 3000 |
| --- | --- |
| Dispersant unit | Hydro MV |
| Pre-dispersant name | Water |
| Dispersant name | Water |
| Pump speed | 1800 rpm |
| Models | General |
| Sensitivity | Normal |
| Particle | Irregular |
| Lower/Upper limit of obscuration | 5% – 20% |
| Background measurement time | 10 s |
| Sample measurement time | 12 s |
| Measurement cycles | 3 |

*Solubility study*

The solubility of IVMT-Rx-4 in different bio-relevant media, pH buffers, and organic solvents was determined via the shake-flask method. In brief, an excess amount of IVMT-Rx-4 was added to 3 mL of various bio-relevant medium including Fasted State Simulated Intestinal Fluid (FaSSIF), Fed State Simulated Intestinal Fluid (FeSSIF), Simulated Intestinal Fluid (SIF), Simulated Gastric Fluid (SGF), pH 1.2 buffer and PBS pH 7.4. In addition, solubility of IVMT-Rx-4 in different solvents including, methanol, ethanol, dichloromethane, acetone, acetonitrile, water, and hexane was determined. The samples were shaken in a plate shaker at 25°C and 800 rpm for 24 hrs. Samples were then filtered using a vacuum manifold and diluted using a 1:1 mixture of water: acetonitrile containing internal standard. The concentration of IVMT-Rx-4 in each filtrate was determined using HPLC methods. Chromatographic separation was performed using a C18 column (2.1 mm × 50 mm, 5 µm, 100 Å) with a mobile phase consisting of 95% water (0.1% formic acid)– 95% acetonitrile (0.1% formic acid), delivered at a flow rate of 0.6 mL/min.

**ADME (Absorption, Distribution, metabolism and excretion) and Pharmacokinetics (ADME/PK) Studies.**

*Log D by flask*

Lipophilicity of IVMT-Rx-4 was determined by the Log D flask method. Sodium Phosphate buffer pH 7.4 (10 mM) was used as the aqueous phase and octanol was used as the organic phase. IVMT-Rx-4 was first dissolved in DMSO (10 µL, 1 mM) and loaded into a 96-well deep well plate (Log D plate), followed by the addition of 500 µL of saturated octanol and 500 µL of saturated phosphate buffer. The sealed plate was incubated at 25ºC with continuous agitation at 2000 rpm for 2 hrs. Samples were then separated by centrifugation, diluted 100-fold with a mixture of H_2_0 and acetonitrile containing internal standard, and analyzed via LC-MS/MS. The Log D value was calculated using the following equation:

Where, DF is the dilution factor.

*Stability in Liver microsomes*

Metabolic stability of IVMT-Rx-4 in liver microsomes from human, monkey, dog, and rodent was evaluated in the presence of cofactors NADPH and UDPGA. Test samples were incubated at 37ºC, and Verapamil was used as a positive control. Samples were drawn from the incubation solution at different timepoints, centrifuged, then diluted with ultra-pure H_2_0 and analyzed via LC-MS/MS. The natural logarithm of the remaining parent drug at each timepoint was plotted against incubation time, and the following equation was used to calculate the in vitro half-life:

*in vitro* t_1/2_ = -(0.693/k), where k is the rate constant.

*Drug-substrate based CYP inhibition*

A cytochrome P450 inhibition assay was conducted to evaluate the inhibitory potential of IVMT-Rx-4 against specific CYP enzymes (CYP1A2, 2B6, 2C8, 2C19, 2A6, 2C9, 2D6, 3A4, and 2E1). IVMT-Rx-4 was incubated with selective probe substrates of each CYP isoform in the presence of human liver microsomes. The formation of metabolite was quantified using LC-MS/MS methods. The extent of inhibition was assessed by calculating the remaining enzyme activity and percent inhibition based on analyte-to-internal standard area ratios, using the following equations:

Area Ratio = Peak Area_Analyte_ / Peak Area_Internal Standard_

Remaining Activity (%) = Area Ratio_test compound_ / Area Ratio_vehicle_ ×100

Inhibition (%) = 100 ̶ Remaining Activity (%)

*Time Dependent Inhibition*

To evaluate the time-dependent inhibition of CYP3A4 by IVMT-Rx-4, a procedure similar to the one described above was employed. IVMT-Rx-4 was preincubated with human liver microsomes in the presence or absence of NADPH to evaluate metabolic activation. Then, the specific CYP3A4 substrate was added, and aliquots were collected at various time points. Samples were then diluted with ultra-pure water and analyzed by LC-MS/MS. Area ratio and remaining Activity (%) were calculated as above and IC50 values was calculated using Excel XLfit 5.3.1.3.

*PXR assay using DPX2 cells*

DPX2 cells were initially cultured in T-75 flasks and maintained at 37°C in a humidified incubator with 5% CO_2_ and 95% relative humidity. Cells were allowed to reach 70-80% confluence prior to subculture. Next, cells were transferred to a culture plate and incubated for an additional 24 hours to allow for attachment and recovery prior to treatment. To assess PXR activation, they were treated with varying concentrations of IVMT-Rx-4. Cell viability and luciferase activity were measured using the CellTiter-Fluor™ Cell Viability Assay and One-GloTM Luciferase Assay System, respectively. Luciferase activity was normalized to viable number and fold activation of PXR was calculated using the following equation:


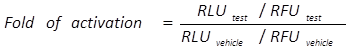


*hERG assay by manual patch clamp assay*

The inhibitory potential of IVMT-Rx-4 on hERG potassium channels was evaluated using a manual patch clamp assay. HEK 293 cells stably expressing hERG (Invitrogen) were cultured in 85% DMEM, 10% dialyzed FBS, 0.1 mM NEAA, 25 mM HEPES, 100U/mL Penicillin-Streptomycin, 5 μg/mL Blasticidin, and 400 μg/mL Geneticin. Baseline hERG current activity was determined using a vehicle control. Subsequently, cells were exposed to different concentrations of IVMT-Rx-4, and changes in hERG-mediated currents were recorded (n=3).

*Mini-Ames assay*

The mini-AMES assay was performed in Pharmaron, Inc. following the standard protocol as developed by the in-house scientist. In this assay, *Salmonella typhimurium* (TA98, TA100, TA1535, TA1537 and WP2 uvrA (pKM101)) were used to evaluate the test article IVMT-Rx-4 (99.7% pure) for its ability to induce reverse mutations at the histidine locus. The assay was conducted in the presence or absence of the S9 mix along with concurrent negative/solvent control and positive controls. Unlimited by the test article solubility, the dose levels tested in the Mini-Ames assay were 1.5, 4, 10, 25, 64, 160, 400 and 1000 μg /well for all five strains. No obvious cytotoxicity was observed at any dose level tested in any tester strains either in the presence or absence of S9 mix.

*Bidirectional Permeability in Caco-2 Cell Line*

To prepare the Caco-2 cell monolayer, 50 µL and 25 µL of cell culture medium (Dulbecco’s modified Eagle’s medium, DMEM) were added to each well of the inserts and reservoir, respectively, within a 96-well HTS Transwell plate (Corning, NY, USA). The plate was incubated at 37 °C and 5% CO_2_ for 1 hr. prior to cell seeding. Caco-2 cells were then diluted to 6.86 x 10^5^ cells/mL using culture medium and 50 µL of cell suspension were seeded into the plate, and incubated at 37 °C, 5% CO_2_, and 95% relative humidity for 14-18 days. During this time, the cells were cultivated with media replacements occurring every other day. Cells were allowed to grow and differentiate and the integrity of the Caco-2 cell monolayer was evaluated by measurement of transepithelial electrical resistance (TEER) using a Millicell Epithelial Volt-Ohm measuring system (Millipore, USA). Caco-2 cell monolayer with TEER values > 230 $\Omega\cdot$cm^2^ were used for experiment to measure the permeability of IVMT-Rx-4.

Prior to the assay, the established monolayer was washed twice with pre-warmed HBSS and incubated at 37 °C for 30 minutes. A bidirectional permeability assay was performed simultaneously using two different plates as described previously (Supplementary Reference: Cui Y, Desevaux C, Truebenbach I, Sieger P, Klinder K, Long A, Sauer A. A Bidirectional Permeability Assay for beyond Rule of 5 Compounds. Pharmaceutics. 2021 Jul 27;13(8):1146. doi: 10.3390/pharmaceutics13081146. PMID: 34452112; PMCID: PMC8400635.): one assessed permeability from the apical to the basolateral side, while the other assessed from the basolateral to the apical side. In brief, IVMT-Rx-4 and control compounds were dissolved in DMSO at 2 mM stock concentration and further diluted with HBSS (25 mM HBSS, pH 7.4) to a working concentration of 10 µM. For apical to basolateral permeability, 108 µL of working solution was loaded into the apical compartment, whereas 308 µL of working solution was added to the basolateral compartment for basolateral to apical permeability. The samples collected from donor and receiver compartment were mixed with 72 µL transport buffer and 240 µL of acetonitrile containing internal standard (IS, 100 nM alprazolam, 200 nM Caffeine and 100 nM tolbutamide), centrifuged at 1000 rpm for 10 minutes. Supernatants were diluted with water and analyzed via LC-MS/MS to determine IVMT-Rx-4 concentrations, using the results to calculate apparent permeabilities and efflux ratio for each of the studies. In doing so, the following equations were used:

Where, P_app_ is apparent permeability (cm/s x 10^-6^), dQ/dt is the rate of drug transport (pmol/second), A is the surface area of the membrane (cm^2^), and D_0_ is the initial donor concentration (nM; pmol/cm^3^). (B-A) indicates basolateral to apical permeation, where (A-B) indicates apical to basolateral.

*Pharmacokinetic studies*

We performed pharmacokinetic studies of IVMT-Rx-4 on four routes of administration including oral, intravenous, intraperitoneal, and subcutaneous. CD1 mice were randomly grouped to 4 groups (n=3 per group). IVMT-Rx-4 was dissolved in a mixture of 10% PEG 400 + 5% DMSO + 85% of 10% HP-β CD. Group 1 received 3 mg/kg of IVMT-Rx-4 via intravenous route; Group 2 received 10 mg/kg of IVMT-Rx-4 via oral route; Group 3 received 10 mg/kg of IVMT-Rx-4 via intraperitoneal route; and Group 4 received 10 mg/kg of IVMT-Rx-4 via subcutaneous route. Further, 20-µL blood samples were collected from a capillary in the retro-orbital plexus at pre-determined time intervals, placed into heparinized tubes, and immediately centrifuged (2500 × g, 15 min, 4 °C). The plasma was separated and kept frozen at −80 °C until analysis.

To measure the plasma concentration of IVMT-Rx-4, a total of 15 µL of each standard or plasma samples were mixed with 200 µL acetonitrile containing dexamethasone as IS for precipitating protein. Then the samples were vortexed for 30 s. After centrifugation at 4000 rpm for 15 min at 4 ºC, the supernatant was diluted 3 times with water.5 µL of the diluted supernatant was injected into the Shimadzu LC/MS/MS system with a Phenomenex Kinetex C18 column (2.1 mm × 50 mm, 5 µm, 100 Å ). The samples were chromatographed using a gradient mobile phase consisting of 95% water (0.1% formic acid)– 95% acetonitrile (0.1% formic acid), delivered at a flow rate of 0.6 mL/min.

**Literatures Cited (Supplementary Documents)**

1. Grembecka J, Cierpicki T, Devedjiev Y, Derewenda U, Kang BS, Bushweller JH, Derewenda ZS. The binding of the PDZ tandem of syntenin to target proteins. Biochemistry. 2006;45(11):3674-83. Epub 2006/03/15. doi: 10.1021/bi052225y. PubMed PMID: 16533050.

2. McNutt AT, Francoeur P, Aggarwal R, Masuda T, Meli R, Ragoza M, Sunseri J, Koes DR. GNINA 1.0: molecular docking with deep learning. Journal of Cheminformatics. 2021;13(1):43. doi: 10.1186/s13321-021-00522-2.

3. Schrodinger Release 2024-4: Glide. New York, NY: Schrodinger, LLC; 2024.
