# Supplementary material for: MDA-9/Syntenin small molecule inhibitor IVMT-Rx-4 blocks prostate cancer bone metastasis
Source: Pharmacol Res. Author manuscript; Available in PMC 2026 Jul 25. (PMC13401508; doi:10.1016/j.phrs.2026.108164)
Supplement: MMC9 [file NIHMS2174343-supplement-MMC9.docx]

**Maji *et al*., Supplementary Table S3**

**Supplementary Table S3: Summary of pharmacokinetic parameters after IV dosing (3 mg/kg body weight)**

| PK parameters |  | Unit | Mouse 1 | Mouse 2 | Mouse 3 | Mean | SD | CV(%) |
| --- | --- | --- | --- | --- | --- | --- | --- | --- |
| Cl_obs | | mL/min/kg | 88.6 | 102.4 | 104 | 98 | 9 | 8.75 |
| T_1/2_ | | h | 0.600 | 0.495 | 0.262 | 0.452 | 0.173 | 38.3 |
| C_0_ | | ng/mL | 2459 | 2347 | 2619 | 2475 | 136 | 5.51 |
| AUC_last_ | | h*ng/mL | 562 | 488 | 478 | 509 | 46 | 9.10 |
| AUC_Inf_ | | h*ng/mL | 564 | 488 | 479 | 510 | 47 | 9.19 |
| AUC__%Extrap__obs | | % | 0.336 | 0.0933 | 0.262 | 0.231 | 0.124 | 54.0 |
| MRT_Inf__obs | | h | 0.380 | 0.285 | 0.236 | 0.300 | 0.074 | 24.5 |
| AUC_last_/D | | h*mg/mL | 187 | 163 | 159 | 170 | 15 | 9.10 |
| V_ss__obs | | L/kg | 2.02 | 1.75 | 1.48 | 1.75 | 0.27 | 15.6 |
